# Oncostatin M upregulates CD73 via the MAPK pathway in keratinocytes to promote an adenosine-dependent anti-inflammatory response in psoriasis

**DOI:** 10.3389/fimmu.2026.1698290

**Published:** 2026-02-06

**Authors:** Caterina Giraulo, Giacomo De Palma, Paola Plaitano, Roberta Esposito, Elva Morretta, Maria Chiara Monti, Christa E. Müller, Carla Cicala, Silvana Morello

**Affiliations:** 1 Department of Pharmacy, University of Salerno, Fisciano, SA, Italy; 2 PhD Program in Drug Discovery and Development, University of Salerno, Fisciano, SA, Italy; 3 Department of Pharmacy, University of Naples “Federico II”, Napoli, NA, Italy; 4 PharmaCenter Bonn, Pharmaceutical Institute, Pharmaceutical and Medicinal Chemistry, University of Bonn, Bonn, Germany

**Keywords:** adenosine, CD73, dermal fibroblasts, inflammation, keratinocytes, oncostatin M, psoriasis

## Abstract

**Introduction:**

Psoriasis is a chronic inflammatory skin condition driven by activated epidermal keratinocytes, dermal fibroblasts, and immune cell infiltrates, which causes tissue injury. The ecto-5′-nucleotidase CD73/adenosine pathway plays a critical role in controlling inflammatory/immune responses, yet it is dysregulated in psoriasis patients. However, the expression and function of this pathway in psoriasis remain poorly explored.

**Methods:**

In this study, we investigated the regulation of CD73 in keratinocytes and examined the anti-inflammatory effects of adenosine in keratinocytes and dermal fibroblasts under psoriatic-like conditions. HaCaT cells and primary normal human epidermal keratinocytes (NHEK) were stimulated with the M5 cytokine cocktail, comprising interleukin (IL)-1α, IL-17A, IL-22, oncostatin M (OSM), and tumor necrosis factor (TNF)-α, to induce a proinflammatory phenotype.

**Results:**

M5-treated keratinocytes release IL-1β, IL-6, and IL-8, as well as the antimicrobial peptide S100A9, and exhibit activation of the signal transducer and activator of transcription (STAT), nuclear factor-kappa B (NF-κB), mitogen-activated protein kinase (MAPK), and phosphoinositide 3-kinase/protein kinase B (PI3K/Akt) pathways. We demonstrated that CD73 is upregulated in inflamed keratinocytes, with OSM identified as a regulator of CD73 expression in a Janus kinase/MAPK-dependent manner. High CD73 expression in inflamed keratinocytes is associated with increased adenosine production. In M5-stimulated keratinocytes, adenosine A_2A_ receptors (A_2A_R) expression is increased, whereas A_2B_R expression is decreased. Functional analyses revealed that an A_2A_R agonist, and to a lesser extent an A_2B_R agonist, reduced IL-8 levels in inflamed keratinocytes. Similarly, M5-treated dermal fibroblasts released IL-1β, IL-6, and IL-8, and exhibited activation of inflammatory signaling pathways. In inflamed dermal fibroblasts, both A_2A_R and A_2B_R were upregulated, and IL-8 release was mitigated by an A_2A_R agonist.

**Discussion:**

In conclusion, these results provide new insights into the mechanisms by which the CD73/adenosine axis can be modulated in psoriatic conditions and may guide the development of effective strategies to mitigate inflammation.

## Introduction

Psoriasis is a chronic inflammatory autoimmune disease that primarily affects the skin. It is characterized by abnormal proliferation and differentiation of keratinocytes, extensive dermal and epidermal infiltration of inflammatory and immune cells—including T cells, dendritic cells, neutrophils, monocytes and macrophages—elevated levels of proinflammatory mediators such as tumor necrosis factor (TNF)-α, interleukin (IL)-12, IL-22, IL-23, IL-17, and chemokines, and increased neoangiogenesis with high levels of growth factors (1, 2). Epidermal keratinocytes critically contribute to the initiation and progression of psoriasis, as they not only proliferate rapidly, causing epidermal hyperplasia [reviewed in (3–5)] but also produce proinflammatory cytokines and antimicrobial peptides (5, 6), which further recruit and activate immune cells, establishing a “feed-forward” inflammatory loop. Dermal fibroblasts play a critical role in psoriatic skin, as they not only contribute to keratinocyte proliferation (7, 8) but are also major producers of the proinflammatory mediators IL-6 and IL-8 in psoriasis lesions, which modulate immune cell function (9). The excessive inflammatory response resulting from the complex network of interactions between immune and stromal cells is associated with tissue damage and disease progression. Therefore, targeting the inflammatory response has the potential to ameliorate the signs of disease in patients.

The CD73/adenosine axis is a well-characterized signaling pathway that plays a crucial role in modulating inflammatory and immune-mediated responses, preventing tissue damage, and restoring homeostasis during prolonged inflammation (reviewed in (10–12). The main source of extracellular adenosine is ATP, which is dephosphorylated to adenosine diphosphate (ADP) and further to adenosine monophosphate (AMP) by the membrane enzyme ecto-enzyme nucleosidetriphosphate diphosphohydrolase-1 (CD39); AMP is then hydrolyzed by CD73, producing adenosine (13). During inflammation and tissue damage, ATP released in high amounts from activated or stressed cells is counterbalanced by adenosine (14). In this context, ATP initiates the inflammatory and immune-mediated responses by stimulating inflammatory and endothelial cells, thereby amplifying the inflammatory process (14). However, at sites of inflammation, excessive ATP may be degraded to adenosine, which strongly inhibits ongoing inflammation and promotes its resolution. The balance between extracellular ATP and adenosine is finely regulated by the expression and activity of nucleotide-degrading enzymes, including CD39 and CD73. In particular, CD73 is the rate-limiting enzyme in the generation of extracellular adenosine, and its expression and activity increase under pathophysiological conditions in response to tissue hypoxia and inflammatory mediators, leading to extracellular adenosine accumulation that acts as a protective brake to limit tissue damage (14).

Adenosine-induced effects are mediated by the receptor subtypes A_1_ (A_1_R), A_2A_ (A_2A_R), A_2B_ (A_2B_R), and A_3_ (A_3_R), which differ in cellular distribution, adenosine affinity, and signal transduction. A_1_R and A_3_R are coupled to G_i/o_ proteins, resulting in a reduction of intracellular cyclic AMP (cAMP), whereas A_2A_R and A_2B_R, coupled to G_s_ proteins, increase intracellular cAMP levels. Additionally, A_2B_R can activate phospholipase C through coupling to G_q_ proteins. The receptors A_1_, A_2A_, and A_3_ exhibit high affinity for adenosine, whereas A_2B_R is a low-affinity receptor (15). At inflammatory sites, adenosine potently inhibits the release of proinflammatory cytokines, promotes the production of the anti-inflammatory cytokine IL-10 from monocytes and granulocytes (16–18), suppresses T-cell responses, including cytotoxicity and cytokine production (19, 20), and induces regulatory T-cell activity (21, 22). These anti-inflammatory and immunomodulatory effects are mediated by the adenosine-generating enzyme CD73 and, among the adenosine receptor subtypes, primarily by A_2A_R (23).

The CD73/adenosine pathway is dysregulated in psoriatic patients and may contribute to psoriasis pathogenesis (24). Overexpression of the A_3_R subtype has been observed in peripheral blood cells from patients with autoimmune disease, such as psoriasis (25), whereas the expression of the receptor subtypes A_2A_ and A_2B_ is dysregulated in psoriatic epidermis compared with normal epidermis (26). In the peripheral blood of patients with *psoriasis vulgaris*, CD73 expression on T-regulatory cells (Treg) is significantly reduced, reflecting a dysfunction that may impair the capacity of these cells to suppress the immune-mediated responses in psoriatic patients (27). To date, based on the role of A_3_R in regulating inflammation (28), an A_3_R agonist is being investigated for the treatment of patients with psoriasis (29), and recently, considerable attention has focused on the development of light-activated A_3_R agonists (30, 31). Promising results have also been obtained with positive allosteric modulators of A_2A_R in a mouse model of skin inflammation (32). Activation of the A_2A_R partially mediates the anti-inflammatory effects of methotrexate, a drug used to treat severe psoriasis, which enhances the release of extracellular adenosine (33, 34). However, although this evidence suggests that the adenosine signaling pathway may represent a potential therapeutic target for inflammatory diseases, including skin inflammation, our understanding of the extent to which adenosine contributes to the pathogenesis of psoriasis remains limited.

In the present study, we investigated whether the adenosine-generating enzyme CD73 is modulated by inflammatory stimuli in keratinocytes under psoriasis-like conditions and examined its potential role in restraining inflammation. Using human keratinocytes exposed to the cytokine cocktail M5 (TNF-α, IL-17A, IL-22, IL-1α, and oncostatin M [OSM]), which models a psoriasis-like environment, we explored the mechanisms regulating CD73 in response to inflammation. We also assessed CD73-dependent AMPase activity, which leads to increased adenosine production and mitigates the inflammatory response in keratinocytes and dermal fibroblasts under psoriasis-like conditions.

## Materials and methods

### Cell cultures

HaCaT cells (passages 13–24), a human immortalized keratinocyte cell line, were cultured in DMEM high-glucose medium supplemented with 10% fetal bovine serum (FBS), penicillin (100 U/mL), and streptomycin (100 µg/mL) (Euroclone, Pero, Italy). Adult primary normal human epidermal keratinocytes (NHEK) were cultured in dermal cell basal medium supplemented with the keratinocyte growth kit (ATCC, Manassas, VA, USA) and used at passages 2–4. BJ cells, a human immortalized dermal fibroblast cell line, were maintained in MEM supplemented with 10% heat-inactivated FBS, penicillin (100 U/mL), streptomycin (100 µg/mL), and 1% nonessential amino acids (Euroclone, Pero, Italy). All cells were incubated at 37°C in a humidified atmosphere with 5% CO_2_.

### Assessment of the psoriatic model *in vitro* and cell treatments

HaCaT cells, NHEK, or BJ cells were stimulated with 10 ng/mL of a proinflammatory cytokine mix, referred to as the M5 cocktail, comprising TNF-α, IL-17A, IL-22, IL-1α, and OSM (PeproTech, Rocky Hill, USA), to mimic psoriatic inflammation (35). Experiments were also performed in HaCaT cells with each cytokine alone—TNF-α, IL-17A, IL-22, IL-1α, or OSM—at a concentration of 10 ng/mL. Dexamethasone (1 µM) (Sigma-Aldrich, St. Louis, MO, USA) was added to HaCaT cells 1 h prior to stimulation with M5 (10 ng/mL) for 24 h (36, 37).

In some experiments, HaCaT cells were treated with ruxolitinib, a Janus kinase (JAK)1/2 inhibitor, at a final concentration of 1 µM (38), 15 min prior to OSM (10 ng/mL) stimulation; with U0126 (500 nM) or LY-294006 (5 µM), selective inhibitors of mitogen-activated protein kinase (MEK)1/2 and phosphoinositide 3-kinase (PI3K), respectively (39); with BSJ-04-122 (5 µM), a selective inhibitor of MAP kinase kinase (MKK)4/7 (40), 2 h before stimulation with OSM (10 ng/mL); or with STATTIC (5 µM), a selective inhibitor of Signal Transducer and Activator of Transcription 3 (STAT3) (41, 42), 30 min prior to OSM (10 ng/mL) stimulation (all from MedChemExpress, Monmouth Junction, NJ, USA).

The A_2A_R agonist CGS21680 (1 µM) or the A_2B_R agonist BAY606583 (1 µM) was added to HaCaT or BJ cells 30 min before stimulation with the M5 cocktail, as described above. Experiments were also performed in the presence of the A_2A_R antagonists ZM241385 (5 µM) or SCH442416 (1 µM) and the A_2B_R antagonists PSB-1115 (100 nM) or PSB-603 (100 nM). All adenosine receptor agonists and antagonists were obtained from Sigma-Aldrich, MO, USA.

### ELISA assay

Levels of inflammatory mediators in cell supernatants collected from HaCaT cells, NHEK, or BJ cells were determined by enzyme-linked immunosorbent assay (ELISA). IL-6, IL-8, and IL-1β levels were measured using the Human Ready-SET-Go ELISA Kit (Thermo Fisher Scientific, Waltham, MA, USA), while S100A9 levels were measured using the Human Precoated ELISA Kit (Elabscience, Houston, TX, USA), according to the manufacturer’s instructions. Data are expressed as picograms per milliliter (pg/mL).

### Immunofluorescence staining

The activation of the nuclear factor-κB (NF-κB), STAT1, STAT3, extracellular signal-regulated kinases p44/42 (ERK)1/2 and PI3K pathways was assessed by immunofluorescence staining for phosphorylated p65 NF-κB (p65NF-κB) (Ser536), pSTAT1 (Tyr701), pSTAT3 (Tyr705), pERK1/2 (Thr202/Tyr204), and pAkt (Ser473). HaCaT cells were seeded on glass coverslips and cultured in the appropriate medium. At the onset of the experiments, cells were treated with the M5 cocktail (10 ng/mL) for 15 min, washed twice with phosphate-buffered saline solution (PBS), fixed in 4% (w/v) paraformaldehyde (PFA), permeabilized with 0.5% (v/v) Triton X-100, and blocked with 0.5% (w/v) bovine serum albumin (BSA; Sigma-Aldrich, MO, USA). Primary antibodies, listed in **Supplementary Table S1**, were applied overnight at 4°C. After two washing steps, cells were incubated with the secondary antibody, goat anti-rabbit IgG AlexaFluor 555 (1:500; Thermo Fischer Scientific, MA, USA), for 2 h at room temperature in the dark. Nuclei were stained with DAPI (1:3,000; Sigma-Aldrich). Coverslips were mounted using antifade mounting medium and examined under a Confocal Laser Scanning Microscope TCS SP8 (software version 3.5.7.23225; Leica Microsystems, Wetzlar, Germany).

### Western blot analysis

Western blot analysis was performed on total whole-cell lysates to detect CD73, oncostatin M receptor (OSMR), and the inflammatory factors p65NF-κB (Ser536), pSTAT1 (Tyr701), pSTAT3 (Tyr705), pERK1/2 (Thr202/Tyr204), pAkt (Ser473), and pJNK (Thr183/Tyr185). CD73 protein expression was analyzed in HaCaT cells and NHEK after 48 h of stimulation with M5, compared with nontreated control cells. The detection of p65NF-κB, pSTAT1, pSTAT3, pERK1/2, and pAkt was performed on total protein extracts from cells treated for 15 min as described above. Whole-cell lysates were prepared using ice-cold radioimmunoprecipitation assay (RIPA) lysis buffer supplemented with a protease inhibitor cocktail and phosphatase inhibitors NaF (5 mM) and Na_3_VO_4_ (1 mM). A total of 25 µg of protein was separated on 10% SDS-PAGE and then transferred to a nitrocellulose membrane. After blocking in 5% (w/v) BSA, membranes were incubated first with the primary antibody, as listed in **Supplementary Table S2**, overnight at 4°C, and then with the secondary antibody—rabbit anti-mouse IgG (H + L) Horseradish peroxidase (HRP)-conjugated (1:25,000; Invitrogen, Carlsbad, CA, USA) or goat anti-rabbit IgG (H + L) HRP-conjugated (1:20,000; Cohesion Biosciences, London, UK)—for 1 h. Signals were detected using the charge-coupled device LAS4000 Imaging System (GE Healthcare Life Sciences, Chicago, IL, USA). Original uncropped images of Western blots are provided in the **Supplementary Material File**.

### Flow cytometry analysis

CD73 expression on HaCaT cells was also determined by flow cytometry after 48 h of cell stimulation, as described above. A total of 1 × 10^6^ cells/mL of HaCaT cells was stained with anti-human CD73 Allophycocyanin (APC) antibody (0.125 µg/test) or APC IgG1K isotype control (0.125 µg/test) (Thermo Fisher Scientific, MA, USA). Samples were acquired using a BD FACScalibur (Becton Dickinson, Franklin Lakes, NJ, USA) and analyzed with the BD CellQuest Pro Software. Data are expressed as relative fluorescence intensity (RFI), calculated as the ratio between the mean fluorescence intensity (MFI) of the target of interest and the MFI of the control isotype.

### RNA extraction and quantitative real-time PCR analysis

Total RNA from HaCaT cells, NHEK, or BJ cells was extracted after 24 h of M5 treatment using TriFast reagent (Euroclone, Pero, Italy) and reverse-transcribed into cDNA with the QuantiTect Reverse Transcription Kit (Qiagen, Hilden, Germany). Quantitative real-time PCR (qRT-PCR) was performed on a QuantStudio™ 5 instrument (Thermo Fisher Scientific, MA, USA) using Luna Universal qPCR Master Mix (New England Biolabs, Ipswich MA, USA).

The primer sequences used for the target gene are:

CD73 (*NT5E*) Fw: 5′-AAAGGACACGAGAGAAAGGAAGG-3′
CD73 (*NT5E*) Rv: 5′-GAAGAAAGAGGACAGAGGCAGAG-3′
A_2A_R (*ADORA2A*) Fw: 5′-CTCCGGTACAATGGCTTGGT-3′
A_2A_R (*ADORA2A*) Rv: 5′-TGGTTCTTGCCCTCCTTTGG-3′
A_2B_R (*ADORA2B*) Fw: 5′-TACTTCCTGGTGTCCCTGGC-3′
A_2B_R (*ADORA2B*) Rv: 5′-GAGGCAGCCGTAGAAGTCAG-3′
The housekeeping gene primer sequences used are:
β-Actin Fw: 5′-CTACAATGAGCTGCGTGTGGC-3′
β-Actin Rv: 5′-CAGGTCCAGACGCAGGATGGC-3′

The relative expression levels of target genes were normalized to β-actin and quantified using the 2^−ΔΔCt^ method.

### Mass spectrometry analysis

AMPase activity in HaCaT cells was analyzed using ultra-high-performance liquid chromatography–electrospray ionization mass spectrometry (UHPLC-ESI-MS). Briefly, HaCaT cells were treated with the M5 cocktail (10 ng/mL) or with TNF-α, IL-17A, IL-22, IL-1α, or OSM at 10 ng/mL, as described above, for 48 h. Cells were then incubated in 100 µL of PBS reaction buffer at 37°C for 10 min. When required, the selective CD73 inhibitors adenosine-5′-α,β-methylene diphosphate (APCP; 100 µM; Sigma-Aldrich, MO, USA) or PSB-12489 (1 µM) (43) (kindly provided by Prof. Müller, University of Bonn, Germany) were added for 30 min at 37°C. The CD73 substrate, [^15^N]adenosine 5′-monophosphate ([^15^N]AMP; 10 µM; Sigma-Aldrich, MO, USA), was added to each sample. To monitor the reaction, 25 µL of the reaction mixture was collected immediately after adding the substrate and again after 120 min. The reaction was quenched by adding an equal volume of ice-cold trichloroacetic acid (TCA; 5% final concentration; Sigma-Aldrich, MO, USA). Samples were then centrifuged at 3,000×*g* for 10 min at 4°C, dried in a Concentrator Plus (Eppendorf, Hamburg, Germany), and resuspended in 25 µL of an aqueous 10 mM ammonium acetate (AmAc; Sigma-Aldrich) solution containing 0.1% acetic acid (AcOH; Sigma-Aldrich). The produced [^15^N]adenosine was quantified using a *Tribrid Orbitrap Mass Spectrometer* coupled to a *VanquishFlex* UHPLC system (Thermo Fisher Scientific, Bremen, Germany). A [^15^N]adenosine calibration curve was also prepared, in the same buffer, ranging from 10 pg/µL to 10 ng/µL.

Briefly, 2 µL of each sample were injected, and chromatographic separation occurred on a Luna Omega Polar 1.6 µm C18 100 Å column (50 mm × 2.10 mm; Phenomenex, Torrance, CA, USA) at a flow rate of 400 µL/min (40°C), using the following gradient: 1 min at 0% B, 1 to 3.5 min to 5% B, 3.5 to 5 min to 95% B, then held at 95% B until 6 min, and returned to 0% B for 4 min of re-equilibration (A: H_2_O, 10 mM AmAc, 0.1% AcOH; B: MeOH, 0.1% AcOH).

The mass spectrometer was operated in MS1 profiling positive scan mode. Full-scan MS spectra were acquired in the Orbitrap analyzer with the following settings: scan range 250–450 m/z, normalized full-scan automatic gain control (nAGC) target at 25% at 30,000 resolution, and maximum injection time of 54 ms. Source parameters were set as follows: spray voltage at 3,500 V, sheet gas at 50 (Arb) and auxiliary gas at 10 (Arb), ion transfer tube temperature at 325°C, and vaporizer temperature at 350°C. [^15^N]adenosine-extracted ion chromatogram was obtained from the raw files using *SkyLine* software, and each peak area was quantified.

### Statistical analysis

Data from at least three independent experiments are presented as the mean ± standard deviation (SD). Statistical analyses were performed using GraphPad Prism 9.0 (GraphPad Software, San Diego, CA, USA), with significance set at *p* < 0.05. For comparisons between two groups, an unpaired Student’s *t*-test or Mann–Whitney *U* test was used. For comparisons involving more than two groups or multiple variables, one- or two-way ANOVA followed by the appropriate *post-hoc* test was applied. Statistical tests and significance are indicated in the figure legends.

## Results

### CD73 is upregulated in psoriatic keratinocytes

**Figure 1:**
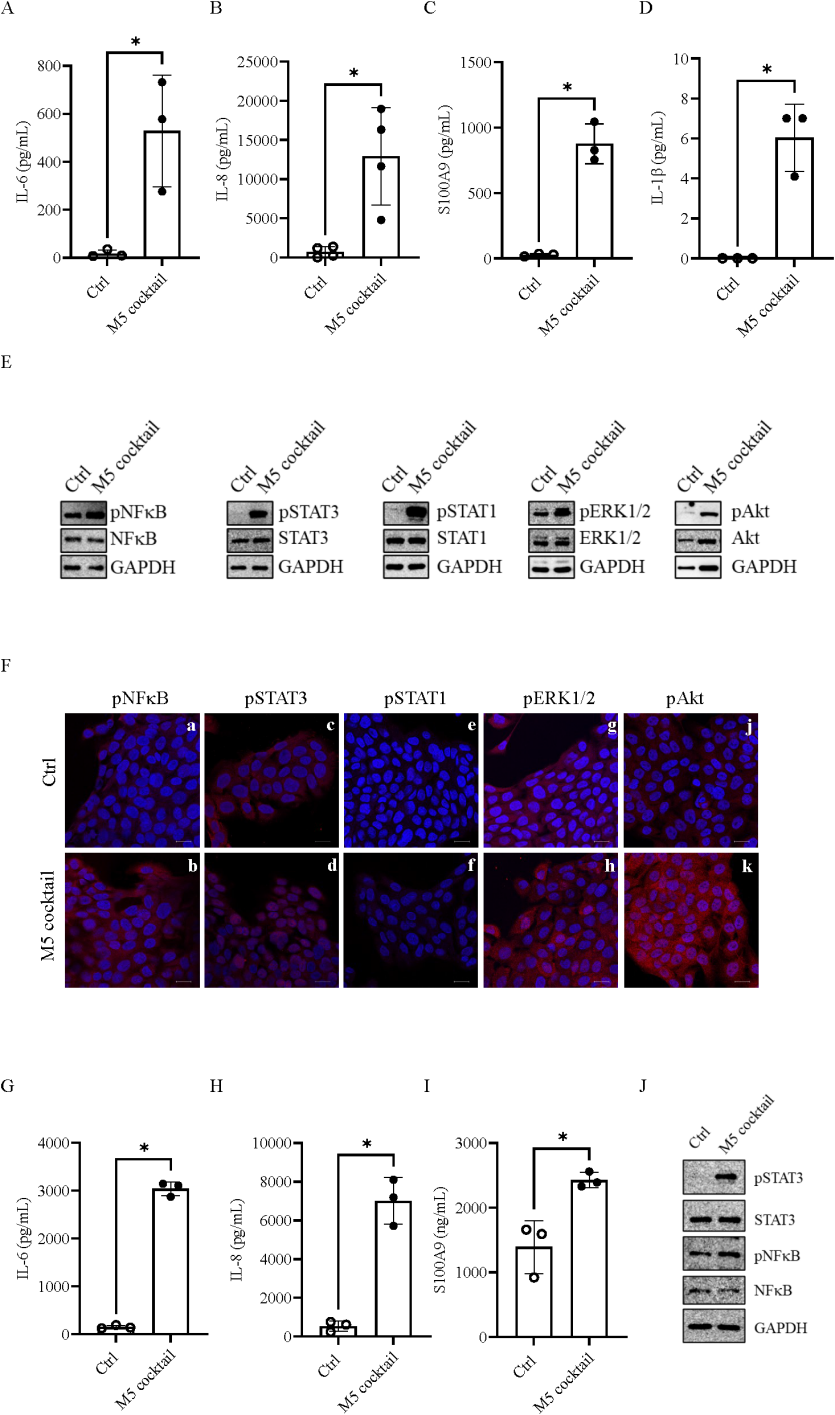
Inflammatory response in keratinocytes stimulated with the M5 cocktail. **(A–C)** IL-6, IL-8, and S100A9 levels, respectively, measured in the supernatant of HaCaT cells collected after 24 h of stimulation with the M5 cocktail (10 ng/mL) by ELISA. **(D)** IL-1β levels measured in the supernatant of HaCaT cells after 48 h of stimulation with M5 cocktail (10 ng/mL) by ELISA. Data are presented as mean ± SD (*n* = 3–4). The *p*-value was obtained using the Mann–Whitney *U* test. ^*^p < 0.05 compared with control cells (Ctrl). **(E)** Representative blots showing the expression of inflammation-associated factors phospho-NF-κBp65, phospho-STAT3, phospho-STAT1, phospho-ERK1/2, and phospho-Akt in HaCaT cells stimulated with the M5 cocktail (10 ng/mL) for 15 min. GAPDH was used as an internal control (*N* = 3). **(F)** HaCaT cells were left untreated or stimulated with the M5 cytokine cocktail (10 ng/mL) for 15 min and stained for phosphorylated NF-κBp65 **(a**, **b)**, STAT3 **(c**, **d)**, STAT1 **(e**, **f)**, ERK1/2 **(g**, **h)**, and Akt **(j**, **k)** (red). Nuclei were counterstained with DAPI (blue). Scale bar = 20 µm. Images were acquired using a × 63/1.4 NA oil immersion objective. **(G–I)** IL-6, IL-8, and S100A9 levels, respectively, measured in the supernatant of normal human epidermal keratinocytes (NHEK) collected after 24 h of stimulation with M5 cocktail (10 ng/mL) by ELISA. Data are presented as mean ± SD (*n* = 3). **(J)** Expression of inflammatory-associated factors phospho-STAT3 and phospho-NF-κBp65 in NHEK cells stimulated with M5 cocktail (10 ng/mL) for 15 min. GAPDH was used as an internal control.

In the present study, we investigated the regulation of CD73 expression in response to psoriasis-related inflammatory stimuli in keratinocytes. For this purpose, we established an *in vitro* model of inflammatory keratinocytes resembling a psoriatic phenotype by treating human HaCaT keratinocytes with the cytokine mixture M5 (10 ng/mL), containing IL-17A, IL-22, OSM, IL-1α, and TNF-α (35). ELISA analysis revealed that treatment with M5 for 24 h significantly upregulated IL-6 (**Figure 1A**), IL-8 (**Figure 1B**), S100A9 (**Figure 1C**) levels, and after 48 h, IL-1β levels (**Figure 1D**). We also assessed p65NF-κB, pSTAT3, pSTAT1, pERK1/2, and pAkt expression in M5-treated HaCaT cells using Western blotting and immunofluorescence after 15 min of stimulation with M5 (10 ng/mL). As shown in **Figures 1E, F**, all these inflammatory signaling pathways were activated in M5-treated HaCaT cells compared with controls. These results confirm that HaCaT cells treated with the M5 cytokine cocktail acquire an inflammatory phenotype that resembles key features of psoriasis, providing a suitable cell culture model to study CD73 regulation in keratinocytes under inflammatory conditions.

The same inflammatory setting was applied to NHEK. When stimulated with M5 (10 ng/mL), these cells released high levels of IL-6, IL-8, and S100A9 (**Figures 1G–I**, respectively), as determined by ELISA of cell supernatants, and expressed pSTAT3 and p65NF-κB (**Figure 1J**), as determined by Western blot analysis.

**Figure 2:**
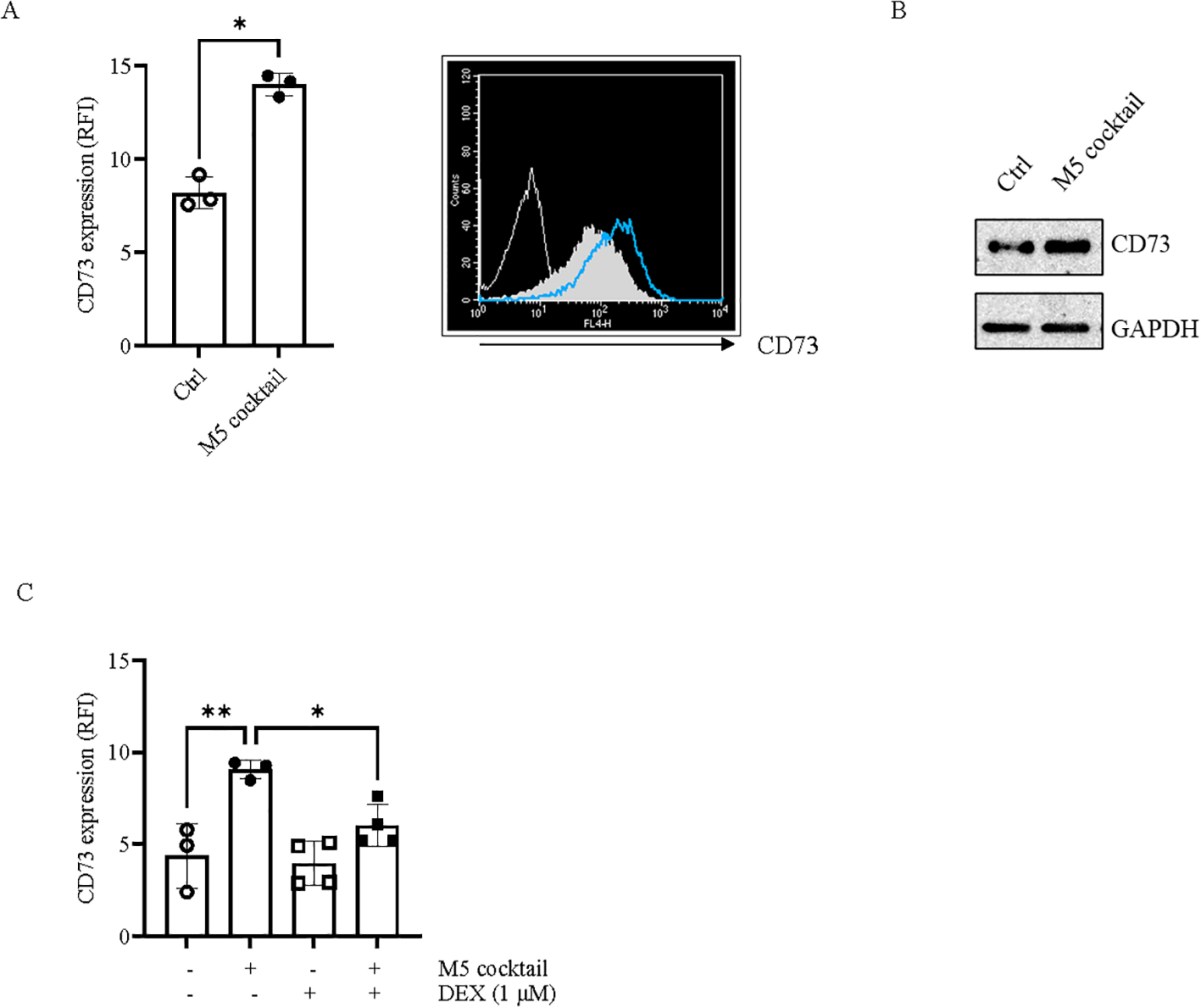
CD73 expression in keratinocytes stimulated with the M5 cocktail. **(A)** CD73 expression was detected by flow cytometry in HaCaT keratinocytes stimulated with the M5 cocktail (10 ng/mL) for 48 h. Data are expressed as relative fluorescence intensity (RFI). Data are mean ± SD (*n* = 3). The *p*-value was obtained using the Mann–Whitney *U* test. ^*^p < 0.05 compared with control cells (Ctrl). Representative histograms showing the isotype control (open white line) or the CD73 expression in cells treated with the M5 cytokine cocktail (blue line) or untreated (filled grey line). **(B)** Representative blot showing CD73 expression in HaCaT cells treated as described in **(A)**. GAPDH was used as an internal control (*N* = 3). **(C)** CD73 expression was detected by flow cytometry in HaCaT cells stimulated or not with M5 cocktail (10 ng/mL) in the presence of dexamethasone (DEX). Dexamethasone (1 µM) was added to HaCaT cells 1 h before M5 stimulation. Data, expressed as specified in **(A)**, are presented as mean ± SD (*n* = 3–4). The *p*-value was obtained using one-way ANOVA followed by Šídák’s multiple comparisons test. ^*^p < 0.05 and ^**^p < 0.01 compared with the indicated controls.

By analyzing CD73 expression at the protein level in HaCaT cells treated with M5, we found that CD73 expression was significantly upregulated compared with control cells, as determined by flow cytometry (**Figure 2A**; **Supplementary Figure S1**) and confirmed by Western blot analysis (**Figure 2B**). Treatment of HaCaT cells with the known anti-inflammatory molecule dexamethasone (1 µM), which significantly reduced the production of proinflammatory cytokine IL-6 as well as the expression of pSTAT3, pSTAT1, pERK1/2, and pAkt induced by M5 stimuli (10 ng/mL) (**Supplementary Figure S2**), markedly inhibited CD73 upregulation (**Figure 2C**), suggesting that CD73 is upregulated in HaCaT cells in response to inflammation.

### Oncostatin M upregulates CD73 expression in keratinocytes

**Figure 3:**
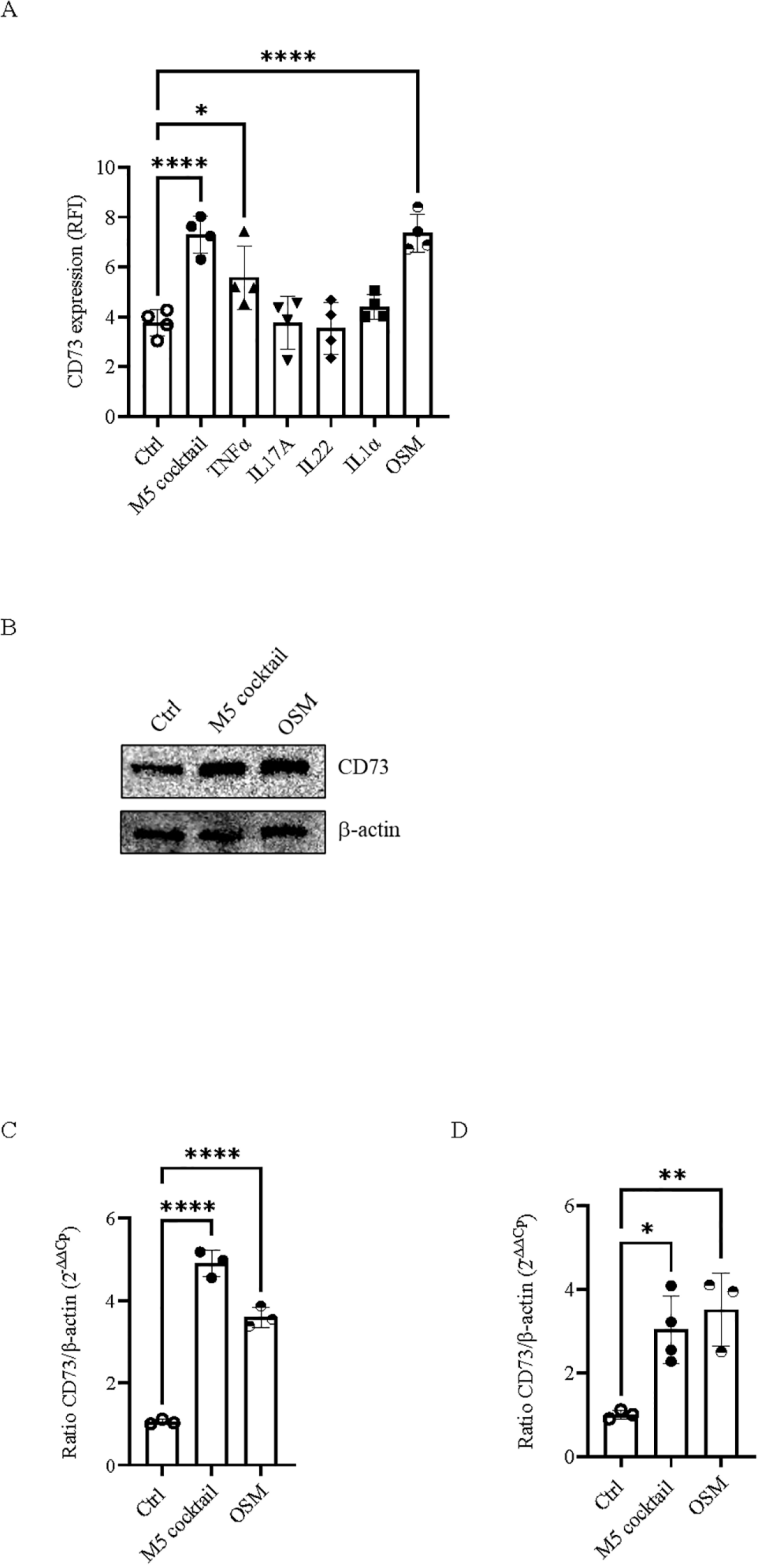
OSM induces CD73 expression in keratinocytes. **(A)** HaCaT cells were treated with an M5 cocktail or TNF-α, IL-17A, IL-22, IL-1α, OSM (10 ng/mL), or medium alone (Ctrl) for 48 h, and CD73 expression was determined by flow cytometry analysis. Data expressed as RFI are mean ± SD (*n* = 4). **(B)** Representative blot showing CD73 expression in NHEK stimulated with the M5 cocktail (10 ng/mL) or OSM (10 ng/mL) for 48 h, compared with control cells. β-Actin was used as an internal control. CD73 mRNA levels in **(C)** HaCaT cells or **(D)** NHEK stimulated for 24 h with the M5 cocktail (10 ng/mL) or OSM (10 ng/mL). Relative CD73 expression level was normalized to β-actin and quantified using the 2^−ΔΔCt^ method. Data are expressed as mean ± SD (*n* = 3–4). The *p*-value was obtained using one-way ANOVA followed by Dunnett’s multiple comparisons test. ^*^p < 0.05, ^**^p < 0.01, and ^****^p < 0.0001, compared with the indicated controls (Ctrl).

To explore the mechanism/s by which the cytokine cocktail M5 induces CD73 expression in keratinocytes, we examined the effects of each cytokine within the M5 combination on CD73 regulation. HaCaT cells were treated with TNF-α, IL-17A, IL-22, IL-1α, or OSM, or with medium alone, for 48 h. Stimulation with TNF-α (10 ng/mL) increased CD73 expression compared with control cells, as determined by flow cytometry analysis (**Figure 3A**). This observation is consistent with previous reports indicating that CD73 expression may be regulated in a TNF-α-dependent manner in malignant cells (44). Notably, OSM (10 ng/mL) strongly increased CD73 protein expression relative to control cells (**Figure 3A**). The other cytokines, including IL-17A, IL-22, or IL-1α (10 ng/mL), did not affect CD73 expression (**Figure 3A**), even when tested at concentrations up to 50 ng/mL (data not shown).

We further analyzed CD73 expression in NHEK. Consistently, CD73 expression was increased in inflamed NHEK stimulated with M5 (10 ng/mL) or OSM (10 ng/mL) compared with control cells (**Figure 3B**).

qRT-PCR analysis confirmed that CD73 expression, which was significantly increased upon M5 stimulation, was also elevated in HaCaT cells stimulated with OSM alone compared with control cells (**Figure 3C**). Similar results were observed in NHEK cells, where M5 or OSM induced CD73 expression compared with control cells (**Figure 3D**). These results demonstrate that CD73 expression in keratinocytes is upregulated by OSM stimulation at both the transcription and protein levels.

### CD73 upregulation induced by OSM in keratinocytes is dependent on the MAPK signaling pathway

Having elucidated that OSM can modulate CD73 expression, we next investigated the signaling pathways involved in the upregulation of CD73 following OSM stimulation in keratinocytes. OSM is a proinflammatory cytokine that can activate STAT proteins and downstream signaling pathways, including the MAPK and PI3K/Akt pathways [reviewed in (44–46)]; these intracellular signaling events are initiated by receptor-associated JAK (47). In keratinocytes, these effects are specifically mediated by the high-affinity type II oncostatin M receptor complex, which contains the gp130 and OSMRβ chains (48, 49). Consistent with these studies, we observed that HaCaT cells stimulated with M5 (10 ng/mL) express the OSMRβ chain (**Supplementary Figure S3**).

**Figure 4:**
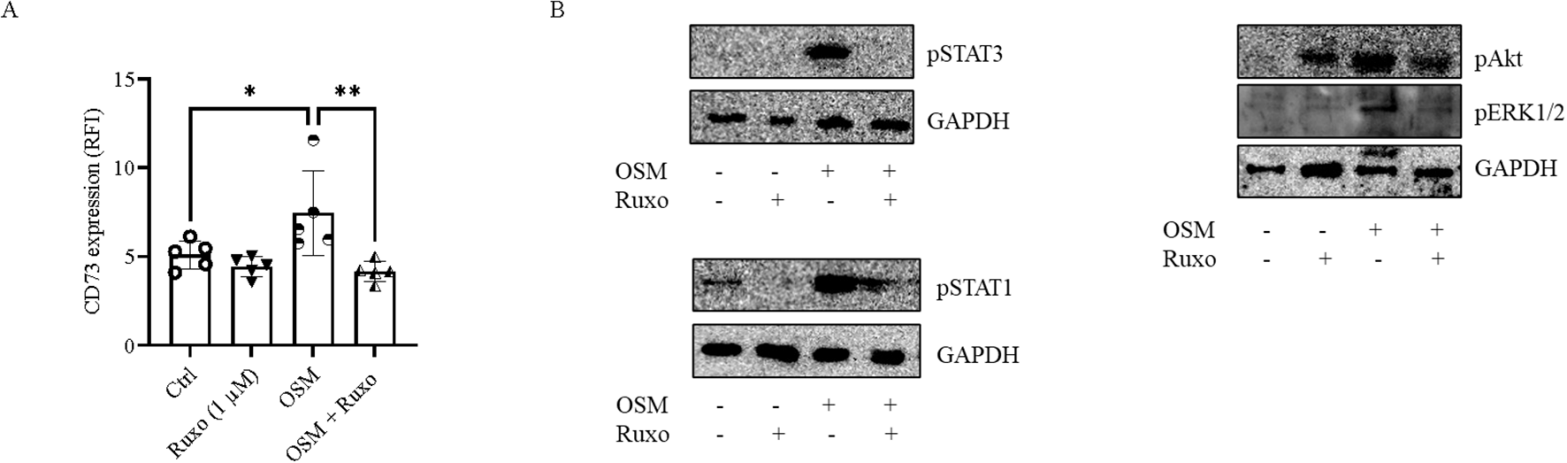
Effect of a JAK inhibitor on OSM-induced CD73 expression in keratinocytes. **(A)** CD73 expression levels measured by flow cytometry in HaCaT cells treated with ruxolitinib (Ruxo) (1 µM) 15 min before OSM (10 ng/mL) stimulation for 48 h. Data, expressed as RFI, are presented as mean ± SD (*n* = 5). The *p*-value was obtained using one-way ANOVA followed by Šídák’s multiple comparisons test. ^*^p < 0.05 and ^**^p < 0.01 compared with the indicated controls (Ctrl). **(B)** Representative blots showing the expression of inflammation-associated factors, including phospho-STAT3, phospho-STAT1, phospho-Akt, and phospho-ERK1/2, in HaCaT cells treated with ruxolitinib (1 µM) for 15 min, followed by OSM (10 ng/mL) treatment for 15 min. GAPDH was used as an internal control. Ctrl, control. *N* = 3.

HaCaT cells were preincubated with the JAK1/2 inhibitor ruxolitinib (1 µM) for 15 min prior to stimulation with OSM (10 ng/mL) for 48 h. Flow cytometry analysis revealed that OSM-induced CD73 expression was significantly abrogated in cells pretreated with ruxolitinib (**Figure 4A**). Western blot analysis revealed that ruxolitinib suppressed OSM-induced phosphorylation of STAT3 and STAT1 (**Figure 4B**), as expected. Phosphorylation of Akt and ERK1/2 in response to OSM was also inhibited in ruxolitinib-pretreated cells (**Figure 4B**).

**Figure 5:**
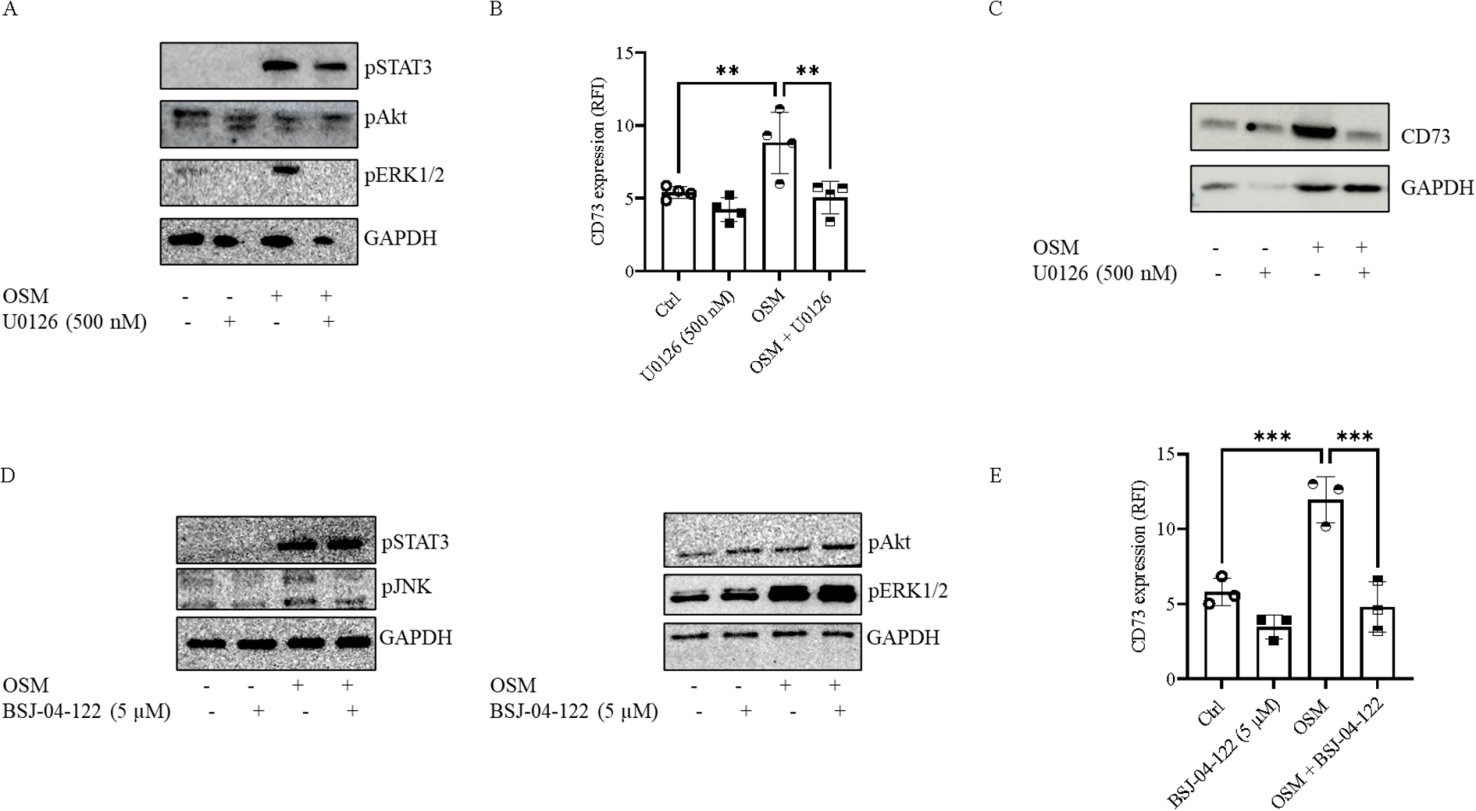
Effect of MAPK inhibitors on OSM-induced CD73 expression in keratinocytes. **(A)** Representative blots showing the expression of inflammation-associated factors phospho-STAT3, phospho-Akt, and phospho-ERK1/2 in HaCaT cells treated with U0126 (500 nM), a MEK 1/2 protein inhibitor, 2 h before the addition of OSM (10 ng/mL) for 15 min. GAPDH was used as an internal control. Ctrl, control. *N* = 3. **(B, C)** CD73 expression determined by flow cytometry and Western blotting, respectively, in keratinocytes treated with U0126 (500 nM) 2 h before the addition of OSM (10 ng/mL) for 48 h. Data in **(B)** are presented as mean ± SD of RFI (*n* = 4). The *p*-value was obtained using a one-way ANOVA test followed by Šídák’s multiple comparisons test. ^**^p < 0.01, compared with the indicated controls. **(D)** Representative blots showing the expression of phospho-STAT3, phospho-JNK, phospho-Akt, and phospho-ERK1/2 in HaCaT cells treated with BSJ-04-122 (5 µM), a MKK4/7 inhibitor, 2 h before the addition of OSM (10 ng/mL) for 15 min. GAPDH was used as an internal control. Ctrl, control. **(E)** CD73 expression determined by flow cytometry in keratinocytes treated with BSJ-04-122 (5 µM) 2 h before the addition of OSM (10 ng/mL) for 48 h. Data in **(E)** are presented as mean ± SD of RFI (*n* = 3). The *p*-value was obtained using a one-way ANOVA test, followed by Šídák’s multiple comparisons test. ^***^p < 0.001, compared with the indicated control.

We then explored whether the activation of MAPK, STAT, or PI3K/Akt signaling pathways contributes to the regulation of CD73 expression induced by OSM, using specific and selective inhibitors of these pathways. The MEK1/2 inhibitor U0126 (500 nM), which significantly blocks ERK1/2 phosphorylation without affecting pSTAT3 or pAkt (**Figure 5A**), was added to the cells 2 h before OSM (10 ng/mL) stimulation for 48 h. U0126 significantly reduced OSM-induced CD73 expression, as determined by flow cytometry (**Figure 5B**) and Western blotting analysis (**Figure 5C**). Similarly, treatment with the MKK4/7 inhibitor BSJ-04-122 (5 µM), which selectively inhibits JNK phosphorylation (**Figure 5D**), added 2 h before OSM (10 ng/mL) stimulation, significantly reduced OSM-induced CD73 expression, as determined by flow cytometry analysis (**Figure 5E**). The STAT3 inhibitor STATTIC (5 µM), added to the cells 30 min before OSM stimulation for 48 h, did not affect OSM-induced CD73 expression (**Supplementary Figures S4A, B**). Western blot analyses showed that STATTIC inhibited STAT3 phosphorylation, whereas the phosphorylation of ERK1/2 and Akt was not affected (**Supplementary Figure S4C**). Pretreatment of cells with the PI3K pathway inhibitor LY-294006 (5 µM) did not inhibit OSM-induced CD73 expression (**Supplementary Figures S5A, B**). We confirmed that LY-294006 suppressed Akt phosphorylation, while STAT3 and ERK1/2 phosphorylation remained unaffected under OSM treatment (**Supplementary Figure S5C**). Together, these results indicate that the activation of the MAP kinase pathways plays a key role in OSM-induced CD73 upregulation in keratinocytes.

### CD73 generates adenosine, which attenuates the inflammatory response in keratinocytes and dermal fibroblasts

**Figure 6:**
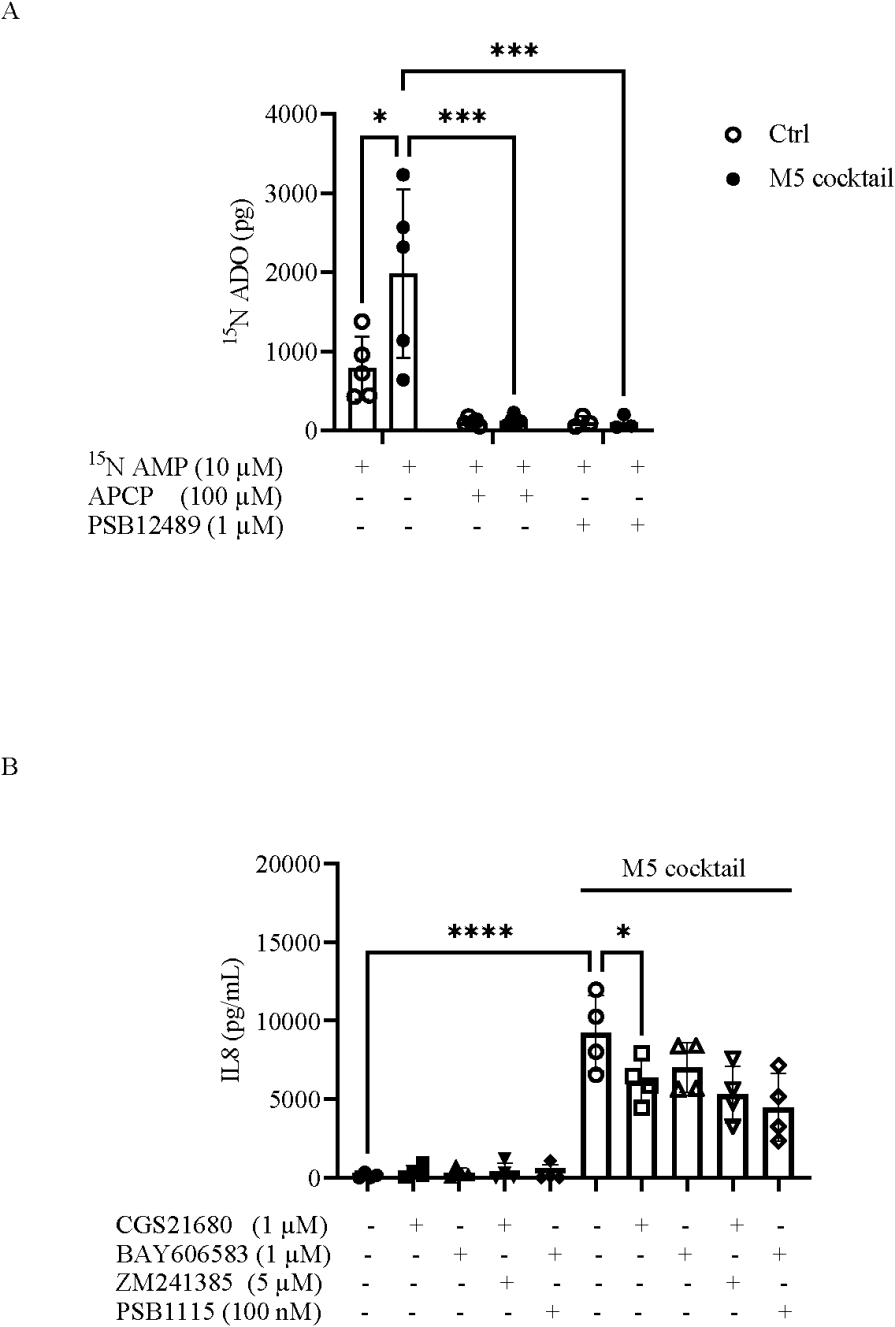
Adenosine production by CD73 and its anti-inflammatory effects in keratinocytes. **(A)** Levels of [^15^N]adenosine ([^15^N]ADO, pg) produced by HaCaT cells in the presence of the substrate [^15^N]AMP (10 µM) following treatment with the M5 cocktail (10 ng/mL) for 48 h, as determined by UHPLC-ESI-MS. Selective CD73 inhibitors, APCP (100 µM) or PSB-12489 (1 µM), were also used to block the AMPase activity mediated by CD73. Data are expressed as mean ± SD (*n* = 5). Ctrl, control. The *p*-value was obtained using two-way ANOVA followed by Šídák’s multiple comparisons test. ^*^p < 0.05; ^***^p < 0.001. **(B)** Levels of IL-8 measured by ELISA in the supernatants of HaCaT cells incubated with CGS21680 (1 µM) or BAY606583 (1 µM), alone or in combination with ZM241385 (5 µM) or PSB-1115 (100 nM), respectively, in the presence or absence of the M5 cocktail (10 ng/mL) for 24 h. Data are expressed as mean ± SD (*n* = 4). The *p*-value was obtained using one-way ANOVA, followed by Šídák’s multiple comparisons test. ^*^p < 0.05 and ^****^p < 0.0001, compared with the indicated controls.

CD73 is the primary enzyme responsible for the generation of extracellular adenosine by dephosphorylating AMP, which is largely derived from ATP degradation (13). To investigate the function of CD73 expressed on keratinocytes in adenosine production, we first measured the AMPase activity in inflamed keratinocytes compared with control cells using [^15^N]AMP as a substrate by mass spectrometry. As shown in **Figure 6A**, [^15^N]adenosine levels were elevated in HaCaT cells stimulated with M5 compared with control cells. To determine whether AMPase activity was dependent on CD73, we also performed experiments using the selective CD73 inhibitors APCP (100 µM) or PSB-12489 (1 µM) (43). In the presence of both CD73 inhibitors, [^15^N]adenosine levels were completely abrogated compared with M5-stimulated cells (**Figure 6A**), indicating that [^15^N]adenosine generation from [^15^N]AMP is entirely dependent on CD73 activity. Although the main analysis focused on CD73 AMPase activity in cells stimulated with the M5 cocktail, we also assessed CD73 activity in cells treated with the individual cytokines composing the cocktail (**Supplementary Figure S6**).

As adenosine is a potent regulator of inflammation and immunity through its receptor subtypes [reviewed in (10, 12)], we next explored the anti-inflammatory effects of adenosine in inflamed M5-treated keratinocytes. For this purpose, we first evaluated the expression profile of the adenosine receptor subtypes A_1_, A_2A_, A_2B_, and A_3_ in HaCaT cells. Consistent with previously published data, we found that the A_2B_R is the most highly expressed adenosine receptor subtype in resting keratinocytes, whereas A_2A_R is expressed at low levels (26). No significant levels of A_1_ and A_3_ receptor messenger RNA (mRNA) were detected (data not shown) (26, 50, 51). Notably, as expected, the expression of these two adenosine receptors, A_2A_ and A_2B_, is modulated in inflamed keratinocytes. Indeed, in M5-stimulated keratinocytes, A_2A_R expression increased compared with control cells (**Supplementary Figure S7A**), whereas A_2B_R expression decreased (**Supplementary Figure S7B**). Using the selective A_2A_R agonist CGS21680 (1 µM), we observed that M5-induced IL-8 release was significantly reduced (**Figure 6B**). Treatment of cells with the A_2B_R agonist BAY606583 (1 µM) also reduced IL-8 levels in M5-stimulated cells, although the effect did not reach statistical significance (**Figure 6B**). However, treatment of cells with the A_2A_R or A_2B_R antagonist ZM241385 (5 µM) or PSB-1115 (100 nM), respectively, did not abrogate the effects induced by the respective A_2A_R agonist CGS21680 or the A_2B_R agonist BAY606583 (**Figure 6B**). Similar results were obtained using the A_2A_R antagonist SCH442416 (1 µM) or the A_2B_R antagonist PSB603 (100 nM) (**Supplementary Figure S8**).

**Figure 7:**
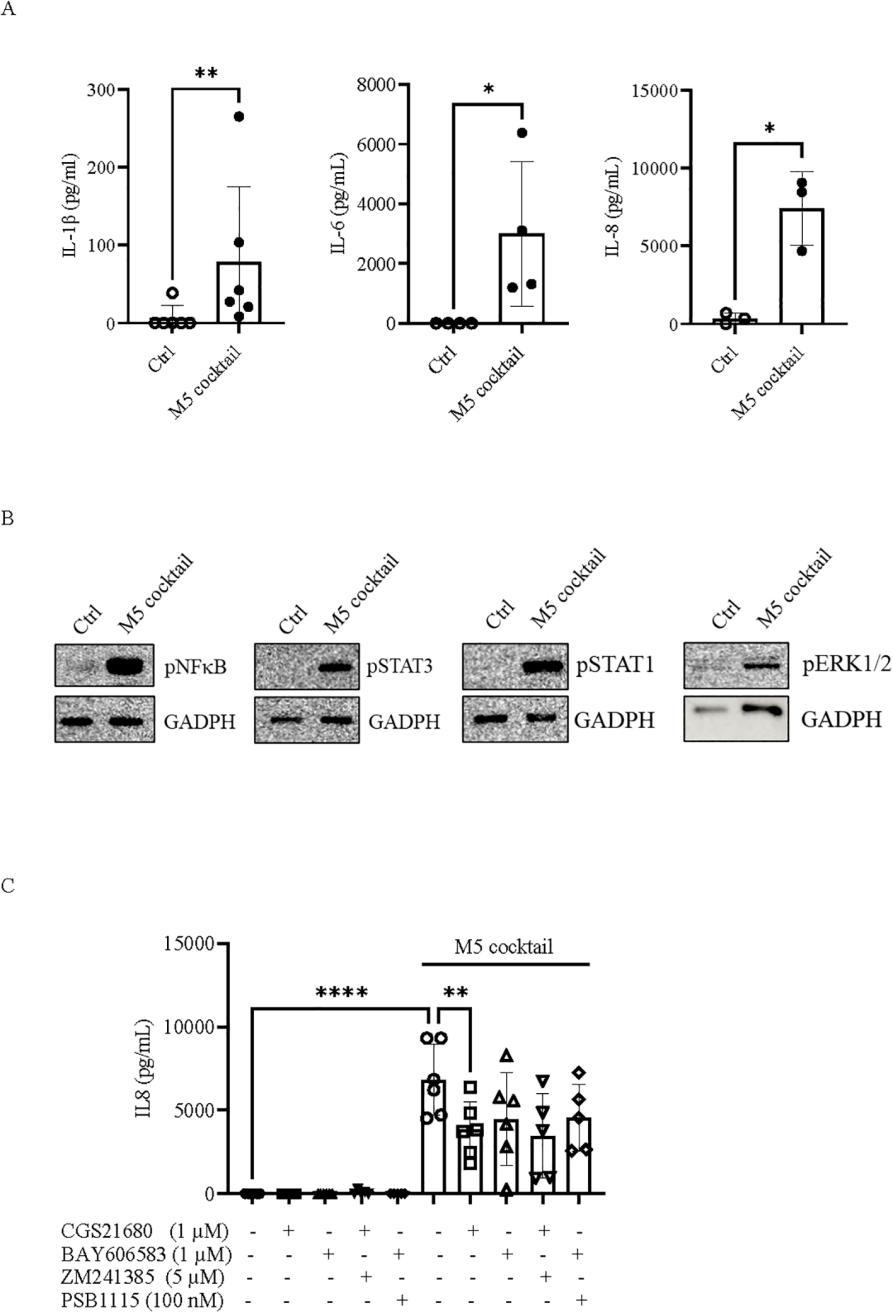
Effect of adenosine receptor agonists in dermal fibroblasts stimulated with the M5 cocktail. **(A)** IL-1β, IL-6, and IL-8 levels measured in the supernatant of human dermal fibroblast BJ cells collected after 24 h of stimulation with the M5 cocktail (10 ng/mL) by ELISA. Data are presented as mean ± SD (*n* = 3–6). The *p*-value was obtained using the Mann–Whitney *U* test. ^*^p < 0.05 and ^**^p < 0.01, compared with the control. **(B)** Expression of inflammation-associated factors phospho-NF-κBp65, phospho-STAT3, phospho-STAT1, and phospho-ERK1/2 in NHEK cells stimulated with the M5 cocktail (10 ng/mL) for 15 min. GAPDH was used as an internal control. Ctrl, control. *N* = 3. **(C)** IL-8 levels measured by ELISA in the supernatants of BJ cells incubated with CGS21680 (1 µM) or BAY606583 (1 µM), alone or in combination with ZM241385 (5 µM) or PSB-1115 (100 nM), respectively, in the presence or absence of the M5 cocktail (10 ng/mL) for 24 h. Data are expressed as mean ± SD (*n* = 6). The *p*-value was obtained using one-way ANOVA followed by Šídák’s multiple comparisons test. ^**^p < 0.01 and ^****^p < 0.0001, compared with the indicated controls.

To further corroborate the potential role of adenosine in restraining the inflammatory response in psoriatic-like conditions, we investigated M5-induced inflammation in dermal fibroblasts. In skin-associated inflammation, dermal fibroblasts play a critical role in exacerbating the inflammatory process and tissue damage by contributing to the production of large amounts of inflammatory cytokines and chemokines (52). In human dermal fibroblasts (BJ cells), M5 treatment (10 ng/mL) induced inflammation, as evidenced by the release of proinflammatory cytokines IL-1β, IL-6, and IL-8 (**Figure 7A**), together with activation of p65NF-κB, pSTAT3, pSTAT1, and pERK1/2 (**Figure 7B**). These results indicate that *in vitro* stimulation of dermal fibroblasts with the psoriasis-related cytokine cocktail M5 can activate an inflammatory response. To evaluate the anti-inflammatory effects of adenosine in inflamed fibroblasts, we analyzed the expression profile of adenosine receptor subtypes. qRT-PCR analysis revealed that A_2A_R, and to a lesser extent A_2B_R, are the major adenosine receptors expressed in BJ cells (data not shown). Notably, in response to inflammation induced by M5 stimulation, the expression of both A_2A_R and A_2B_R increased compared with control cells (**Supplementary Figure S9**), suggesting that adenosine receptor subtypes A_2A_ and A_2B_ are also dysregulated in dermal fibroblasts under psoriatic-like conditions. A_1_ and A_3_ receptor mRNAs were present at very low or undetectable levels (data not shown). Based on these findings, cells were treated with CGS21680 (1 µM) or BAY606583 (1 µM), A_2A_R and A_2B_R agonists, respectively, prior to M5 stimulation, and IL-8 levels were measured in cell supernatants. As shown in **Figure 7C**, treatment of cells with the A_2A_R agonist CGS21680 significantly reduced IL-8 release triggered by M5 stimulation, whereas cells treated with BAY606583 showed a trend toward reduced IL-8 levels. Unexpectedly, the inhibitory effect of CGS21680 on IL-8 release was not abrogated by the A_2A_R antagonist ZM241385 (**Figure 7C**).

Altogether, these results show that increased CD73 expression in inflamed keratinocytes is associated with elevated adenosine production. The expression of the adenosine receptor subtypes A_2A_ and A_2B_—primarily expressed on keratinocytes and dermal fibroblasts—is dysregulated in response to inflammatory psoriatic-related cytokines. Specifically, A_2A_R expression is increased in both cell types during inflammation, whereas A_2B_R expression is reduced in keratinocytes but increased in dermal fibroblasts stimulated with the M5 cocktail. Both A_2A_R and A_2B_R agonists exhibit anti-inflammatory effects *in vitro* in keratinocytes and in dermal fibroblasts activated by the psoriatic-related cytokine cocktail M5.

## Discussion

In this study, we demonstrate that CD73 expression is significantly upregulated in keratinocytes under psoriatic-like conditions and identify OSM as a regulator of CD73 expression via a JAK/MAPK-dependent pathway. The overexpression of CD73 in inflamed keratinocytes is associated with increased adenosine production, a natural brake on inflammation. Furthermore, in response to psoriasis-like inflammation, the expression profiles of A_2A_R and A_2B_R in keratinocytes and dermal fibroblasts are dysregulated. Stimulation of A_2A_R significantly reduces the release of the proinflammatory cytokine IL-8 in keratinocytes and dermal fibroblasts activated by the psoriatic-related cytokine cocktail M5.

Keratinocytes are the major cell population in the epidermis. In psoriasis, proinflammatory cytokines—such as TNF-α, IL-6, IL-1β, OSM, IL-20, IL-22, and IL-17A—are potent modulators of keratinocytes. They synergistically activate these cells, which then rapidly proliferate, differentiate aberrantly, and produce inflammatory cytokines and antimicrobial peptides that further stimulate and activate immune cells in the skin lesions, creating a positive feedback loop (5, 53, 54). In the present study, the M5 cytokine cocktail, containing psoriasis-related inflammatory mediators—including TNF-α, IL-1α, IL-17A, IL-22, and OSM—was used to establish a psoriatic-like cellular model (35). Cells stimulated with M5 acquire a psoriatic proinflammatory phenotype, releasing cytokines and antimicrobial peptides such as IL-6, IL-8, IL-1β, and S100A9. Moreover, keratinocytes, in response to psoriasis-associated inflammatory cytokines, display activation of STAT3, NF-κB, and MAP kinases, which are the major signaling pathways involved in psoriasis (5). During the inflammatory process, keratinocytes in the epidermis respond to tissue damage or cell death by releasing ATP (51), which can be hydrolyzed to ADP and then to AMP by the membrane enzyme CD39. AMP is subsequently hydrolyzed by CD73, producing adenosine (13) to limit excessive inflammation and restore homeostasis (55). Although CD39 is weakly expressed in keratinocytes, as confirmed by our experimental observations (data not shown) (56–59), very little is known about the regulation of CD73 in inflamed and activated keratinocytes. Previous studies have demonstrated the presence of CD73 on human epidermal keratinocytes (58). Here, we demonstrate that in both immortalized keratinocytes and primary NHEK, CD73 expression is upregulated in response to the inflammatory stimulus M5. By analyzing the mechanisms through which the psoriatic-like cytokine cocktail M5 modulates CD73 expression on keratinocytes, we found that among the cytokines contained in the M5 cocktail, OSM alone strongly upregulated CD73 expression. TNF-α stimulation slightly enhanced CD73 expression, whereas IL-1α, IL-22, and IL-17A had no effect. Previous reports indicate that TNF-α is a positive transcriptional regulator of the *NT5E* gene, which encodes CD73, via NF-κB in human colorectal cancer cells (44). Here, we found that TNF-α can modulate CD73 expression in keratinocytes, as expected. Importantly, to the best of our knowledge, we report for the first time that OSM is a key mediator of CD73 induction in keratinocytes. OSM is a proinflammatory cytokine belonging to the IL-6 family, first detected in psoriatic skin in 1998 (60). In skin inflammation, OSM plays a critical role in sustaining inflammation and the immune response, as it is a potent activator of epidermal keratinocytes (49). In keratinocytes expressing OSMR, we observed that OSM promotes phosphorylation of STAT-1, STAT-3, ERK1/2, and Akt. These results are consistent with previous data showing that, in human keratinocytes, OSM activates the STAT, PI3K, and MAPK pathways through its type II OSM receptor complex (48, 49, 61). These events are initiated by receptor-associated Janus kinases 1/2 (61). We further demonstrated that pretreatment of cells with the JAK inhibitor ruxolitinib abrogated the activation of all these signaling events in response to OSM and inhibited CD73 overexpression. When we analyzed the effects of the commercially available selective STAT3 inhibitor STATTIC, we observed that the upregulation of CD73 in response to OSM was not affected, suggesting that STAT3 does not significantly contribute to the regulation of CD73 expression triggered by OSM in keratinocytes. However, we cannot rule out the possible involvement of other STAT family members, such as STAT1 or STAT5. Given the complexity and potential redundancy of STAT signaling in inflammatory pathways, additional studies are needed to explore this aspect further. The PI3K pathway inhibitor LY-294006 also failed to affect the upregulation of CD73 in response to OSM. Notably, pretreatment of cells with the MEK1/2 inhibitor U0126, which selectively abrogates ERK1/2 phosphorylation in keratinocytes in response to OSM, or with the MKK4/7 inhibitor, which selectively inhibits JNK activation, reduced the OSM-induced upregulation of CD73. These results suggest that CD73 is upregulated in keratinocytes in response to OSM via MEK-ERK and JNK signaling. Previous studies have shown that MAPK pathway inhibitors reduce the protein and mRNA levels of CD73 in human malignant cells (62–66). Here, using a pharmacological approach, we confirmed that the MAPK pathway is involved in the upregulation of CD73 in response to inflammatory psoriatic-like stimuli in keratinocytes.

We found that CD73 on keratinocytes is functionally active in producing adenosine. Specifically, AMPase activity is elevated in cells stimulated with the M5 cocktail compared with control cells, as well as in cells treated with OSM (10 ng/mL) or TNF-α (10 ng/mL), suggesting that the increased adenosine production is associated with upregulated CD73 expression.

Considering the well-established role of adenosine in mediating anti-inflammatory and immunoregulatory effects, it is likely that, in response to an inflammatory insult, upregulation of CD73—and the consequent increase in adenosine production—occurs as an adaptive mechanism to mitigate excessive damage induced by inflammation. Supporting the functional relevance of elevated extracellular adenosine in mediating anti-inflammatory effects and reducing leukocyte recruitment to sites of inflammation, clinical evidence indicates that the beneficial effects of methotrexate in severe psoriasis are dependent, at least in part, on extracellular adenosine accumulation (67). Furthermore, MTX has been shown to restore CD73 expression on Th1.17 (effector T cells) in psoriatic patients (68). The anti-inflammatory effects of adenosine are primarily mediated by the A_2A_R and/or A_2B_R subtypes (11). In line with previous reports, we confirmed that resting keratinocytes express the A_2B_R subtype and, to a lesser extent, the A_2A_R subtype, whereas the expression of the A_1_R and A_3_R subtypes is negligible (26, 50, 51). In M5-stimulated keratinocytes, we observed dysregulated expression of A_2A_R and A_2B_R compared with control cells: A_2B_R expression was decreased, whereas A_2A_R expression was enhanced. Our data on the altered expression profile of A_2A_R and A_2B_R in keratinocytes under psoriasis-like conditions are consistent with previous observations in human epidermis samples from patients’ biopsies, which showed reduced A_2B_R expression and increased A_2A_R expression in psoriatic epidermis compared with healthy epidermis (26). In a previous study, Hinz et al. reported that A_2A_R can be blocked by A_2B_R; consequently, upregulation of A_2A_R and/or downregulation of A_2B_R could potentiate the anti-inflammatory effect of A_2A_R activation (69).

Examining the role of adenosine in regulating the inflammatory response of M5-stimulated HaCaT keratinocytes, we found that treatment with selective agonists of A_2A_R and A_2B_R effectively reduced IL-8 release induced by M5 stimulation. Interestingly, this effect was not abrogated by their pharmacological blockade of A_2A_R and A_2B_R. The inhibition of IL-8 release by A_2A_R and A_2B_R agonists in keratinocytes activated with the protein kinase C activator TPA has previously been reported to occur through a mechanism independent of adenosine A_2A_R and/or A_2B_R (26). Similar observations have been made in other cell types, including macrophages and neutrophils (17, 70), where the response depends instead on membrane phosphate activation. Here, based on our data, we speculate that the employed A_2A_R and A_2B_R agonists may act through a mechanism independent of adenosine receptors. However, this remains an open question, and future studies will be required to clarify the underlying mechanisms. In this context, genetic silencing approaches (e.g., siRNA or CRISPR-based strategies) could provide more direct evidence and help validate receptor-specific pathways.

The regulatory role of adenosine in keratinocyte inflammation *in vitro* prompted us to evaluate the effects on the inflammatory response in dermal fibroblasts stimulated with the psoriasis-related cytokine cocktail M5. Dermal fibroblasts, together with keratinocytes, play an important role in producing various cytokines and growth factors in psoriasis. For example, the expression of certain cytokines, such as IL-6 and IL-8, is higher in psoriatic fibroblasts than in psoriatic keratinocytes (9), suggesting that fibroblasts can critically contribute to dysregulating the cytokine profile in psoriatic lesions. In this study, we observed that M5 stimulation of dermal fibroblasts induces an inflammatory response, characterized by high levels of IL-6, IL-8, and IL-1β. By analyzing the adenosine receptor expression profile in human dermal fibroblasts (BJ cells), we found that these cells express A_2A_R and A_2B_R, whereas the A_1_R is expressed at very low levels, and A_3_R mRNA is undetectable. Notably, we provide evidence that both A_2A_R and A_2B_R are dysregulated in dermal fibroblasts stimulated with psoriasis-related cytokines, as they were upregulated in response to M5 stimulation. These findings indicate that the expression of A_2A_R and A_2B_R in a psoriatic-like condition may be altered in a cell type-specific manner, reflecting either enhanced or reduced responsiveness to adenosine in initiating downstream signaling events. In dermal fibroblasts, stimulation of A_2A_R with its agonist was observed to reduce the IL-8 release triggered by M5 stimulation. However, similar to observations in keratinocytes, the anti-inflammatory effect of the A_2A_R agonist in dermal fibroblasts was not reversed by the A_2A_R antagonist. As with keratinocytes, we can only speculate that this mechanism is receptor-independent, which remains to be verified. Nonetheless, these results indicate that the A_2A_R agonist CGS21680 exerts beneficial effects in reducing IL-8 levels from activated dermal fibroblasts *in vitro* under psoriatic-like conditions. IL-8 is a proinflammatory cytokine whose levels have been found be elevated in psoriatic epidermis [reviewed in (64)] and positively correlated with psoriasis severity (71). Increased IL-8 expression is associated with high neutrophil infiltration, which can amplify the inflammatory circuit in skin lesions and promote resistance to psoriasis treatment (72). Despite the encouraging nature of these findings, the extent to which CD73-derived adenosine can mitigate the full spectrum of inflammatory mediators released by keratinocytes and dermal fibroblasts, within the complex network interactions between infiltrating immune cells and tissue-resident cells in psoriasis, requires further investigations, including *in vivo* studies. This consideration is particularly relevant given the potential cell-specific alterations of adenosine receptor subtypes. Furthermore, validation in patient-derived samples from individuals with psoriasis will be essential to confirm the clinical relevance of the CD73/adenosine axis.

## Conclusions

In conclusion, we have identified a novel mechanism regulating CD73 expression in keratinocytes *in vitro* under psoriatic-like conditions. In particular, OSM upregulates CD73 expression in keratinocytes via a JAK/MAP kinase-dependent manner. The overexpression of CD73 in inflamed keratinocytes is associated with increased adenosine release, which mitigates inflammation in both keratinocytes and dermal fibroblasts exposed to psoriatic-related cytokines. These findings support the critical role of the CD73/adenosine pathway in psoriatic conditions and may be helpful in the development of effective anti-inflammatory therapies.

## Data Availability

The raw data supporting the conclusions of this article will be made available by the authors, without undue reservation.
