# Supplementary material for: Oncostatin M upregulates CD73 via the MAPK pathway in keratinocytes to promote an adenosine-dependent anti-inflammatory response in psoriasis
Source: Front Immunol. 2026 Feb 6;17:1698290. doi: 10.3389/fimmu.2026.1698290 (PMC12920598; doi:10.3389/fimmu.2026.1698290)
Supplement: Supplementary file 1 [file DataSheet1.pdf]

Oncostatin M upregulates CD73 via the MAPK pathway in keratinocytes to promote an adenosine-dependent anti-inflammatory response in psoriasis.

Caterina Giraulo[1,2], Giacomo De Palma[3], Paola Plaitano[1], Roberta Esposito[1], Elva Morretta[3], Maria Chiara Monti[3], Christa Müller[4], Carla Cicala[3], Silvana Morello[1]*

Affiliation

[1]Department of Pharmacy, University of Salerno, Fisciano (SA), Italy

[2]PhD Program in Drug Discovery and Development, University of Salerno, Fisciano (SA), Italy

[3]Department of Pharmacy, University of Naples "Federico II", Napoli (NA), Italy

[4]PharmaCenter Bonn, Pharmaceutical Institute, Pharmaceutical & Medicinal Chemistry, University of Bonn, Bonn, Germany.

Corresponding author address: smorello@unisa.it.

Supplementary Tables

Supplementary table 1 – *List of primary antibodies used for immunofluorescence analysis*

Primary antibody	Working concentration	Clone	Host	Source
pNFκB	1:2000	93H1	Rabbit	Cell Signaling Technology, Beverly, MA, USA
pSTAT3	1:2000	D3A7	Rabbit	Cell Signaling Technology, Beverly, MA, USA
pSTAT1	1:1000	58D6	Rabbit	Cell Signaling Technology, Beverly, MA, USA
pERK1/2	1:1000	197G2	Rabbit	Cell Signaling Technology, Beverly, MA, USA
pAkt	1:1000	D9E	Rabbit	Cell Signaling Technology, Beverly, MA, USA

Supplementary table 2 – *List of primary antibodies used for Western blotting analysis*

Primary antibody	Working concentration	Clone	Host	Source
pNFκB	1:2000	93H1	Rabbit	Cell Signaling Technology, Beverly, MA, USA
NFκB	1:2000	L8F6	Mouse	Cell Signaling Technology, Beverly, MA, USA
pSTAT3	1:2000	D3A7	Rabbit	Cell Signaling Technology, Beverly, MA, USA
STAT3	1:2000	D3Z2G	Rabbit	Cell Signaling Technology, Beverly, MA, USA
pSTAT1	1:1000	58D6	Rabbit	Cell Signaling Technology, Beverly, MA, USA
STAT1	1:1000		Rabbit	Proteintech, Rosemont, Illinois, USA
pERK1/2	1:1000	197G2	Rabbit	Cell Signaling Technology, Beverly, MA, USA
ERK1/2	1:1000	137F5	Rabbit	Cell Signaling Technology, Beverly, MA, USA
pAkt	1:1000	D9E	Rabbit	Cell Signaling Technology, Beverly, MA, USA
Akt	1:1000	11ET	Rabbit	Cell Signaling Technology, Beverly, MA, USA
pJNK	1:1000	81E11	Rabbit	Cell Signaling Technology, Beverly, MA, USA
OSMR	1:1000	A874	Mouse	Elabscience, Texas, USA

CD73	1:2000	D7F9A	Rabbit	Cell Signaling Technology, Beverly, MA, USA
GAPDH	1:5000		Mouse	Cohesion Biosciences, London, UK
β-actin	1: 2000		Rabbit	Elabscience, Texas, USA
β-tubulin	1:5000		Mouse	Cohesion Biosciences, London, UK

Supplementary Figures

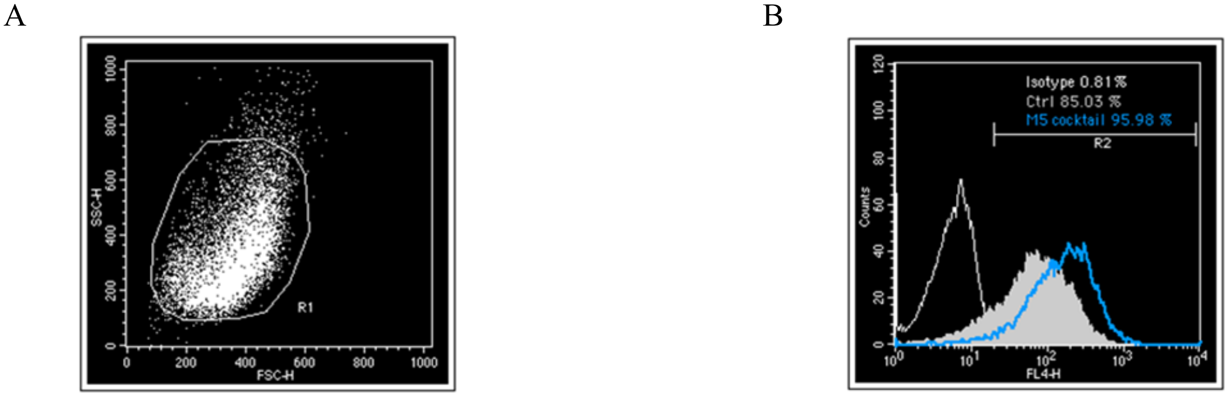

Supplementary figure 1. (A) Gating strategy for HaCaT cells analyzed by flow cytometry, viable cells were selected (R1) and analyzed for the expression of CD73. (B) The panel shows the percentage of CD73-positive cells (R2) calculated within the gate (R1).

A

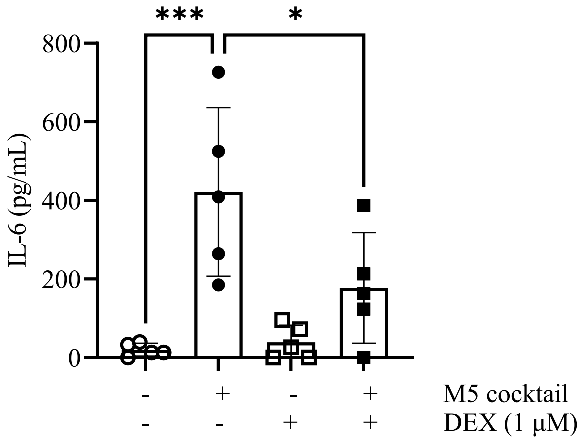

B

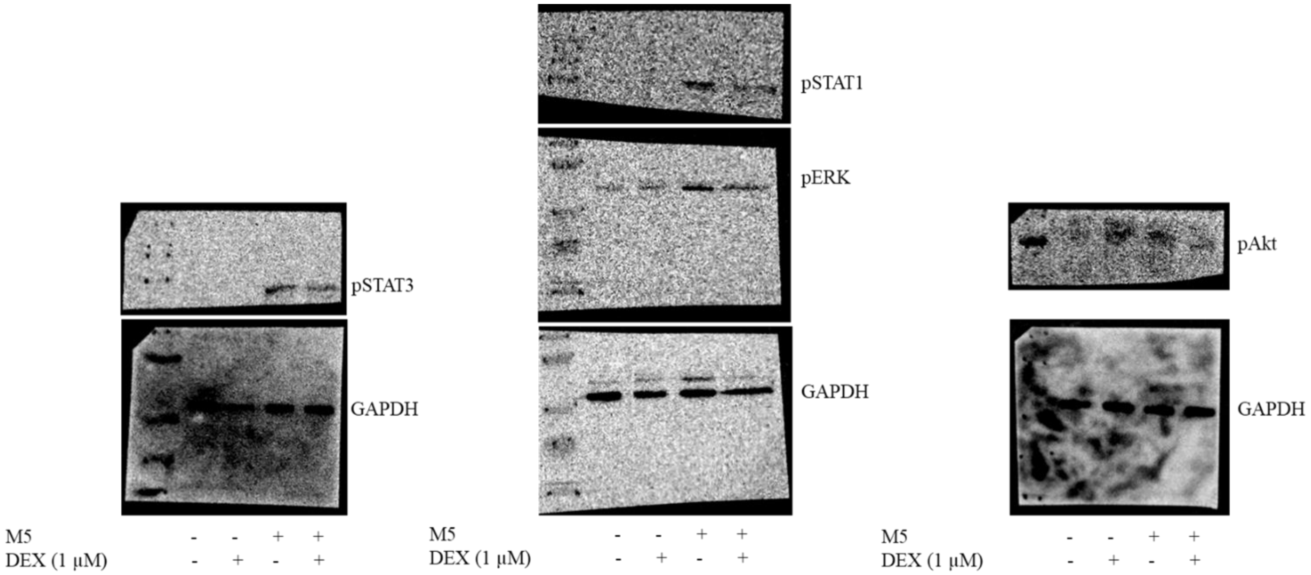

Supplementary figure 2. (A) HaCaT cells were pretreated with dexamethasone (1 µM) (DEX) 1 h before stimulation with M5 (10 ng/mL) for 24 h. IL-6 levels were detected in the supernatant of cells by ELISA assay and expressed as mean ± s.d., n = 5. P value is obtained from one-way ANOVA test, followed by Šídák's multiple comparisons test. *P<0,05, P<0,001, compared with the indicated controls. (B) Representative blots showing the expression of inflammatory-associated factors phospho-STAT3, phospho-STAT1, phospho-ERK1/2 and phospho-Akt in HaCaT cells treated with DEX (1 µM) for 1 h and then with M5 cocktail (10 ng/mL) for 24 h. GAPDH was used as an internal control. Ctrl, control.

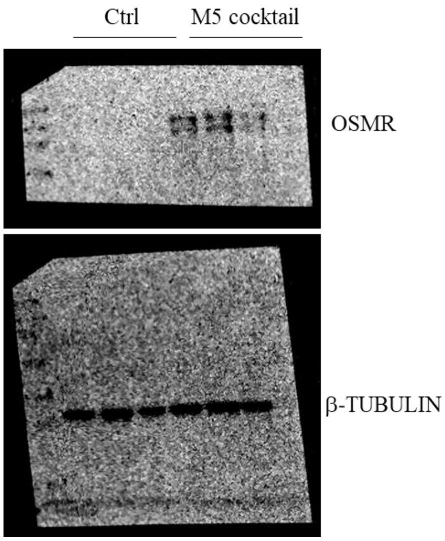

Supplementary figure 3. Representative blots showing the expression of oncostatin M receptor (OSMR) in HaCaT cells treated with M5 cocktail (10 ng/mL) for 48 h compared to control cells (Ctrl). β-tubulin was used as an internal control.

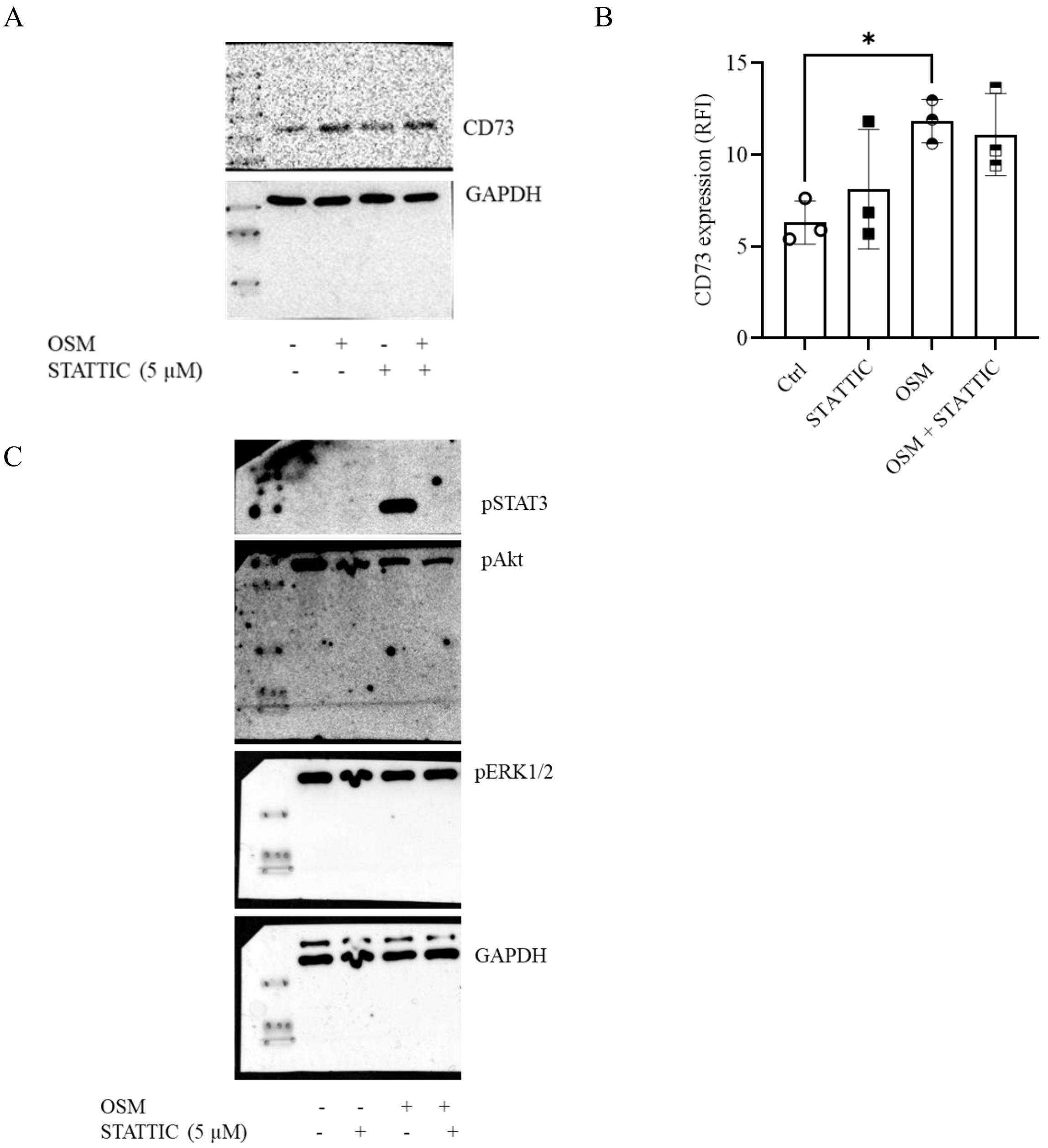

Supplementary figure 4. (A) Representative blot showing the expression of CD73 in cells treated with the STAT3 inhibitor STATTIC (5 µM) 30 min before stimulation with OSM (10 ng/mL) for 48 h. (B) Expression of CD73 determined by flow cytometry in keratinocytes treated with STATTIC (5 µM) 30 min before adding OSM (10 ng/mL) for 48 h. Data are mean ± s.d. as RFI, n = 3. P value is obtained from one-way ANOVA test, followed by Šídák's multiple comparisons test. *P<0.5, compared with the indicated controls. (C) Representative blots showing the expression of phospho STAT3, phospho ERK1/2 and phospho Akt in HaCaT cells treated with the STAT3 inhibitor STATTIC (5 µM) 30 min before stimulation with OSM (10 ng/mL) for 15 min. GAPDH was used as an internal control.

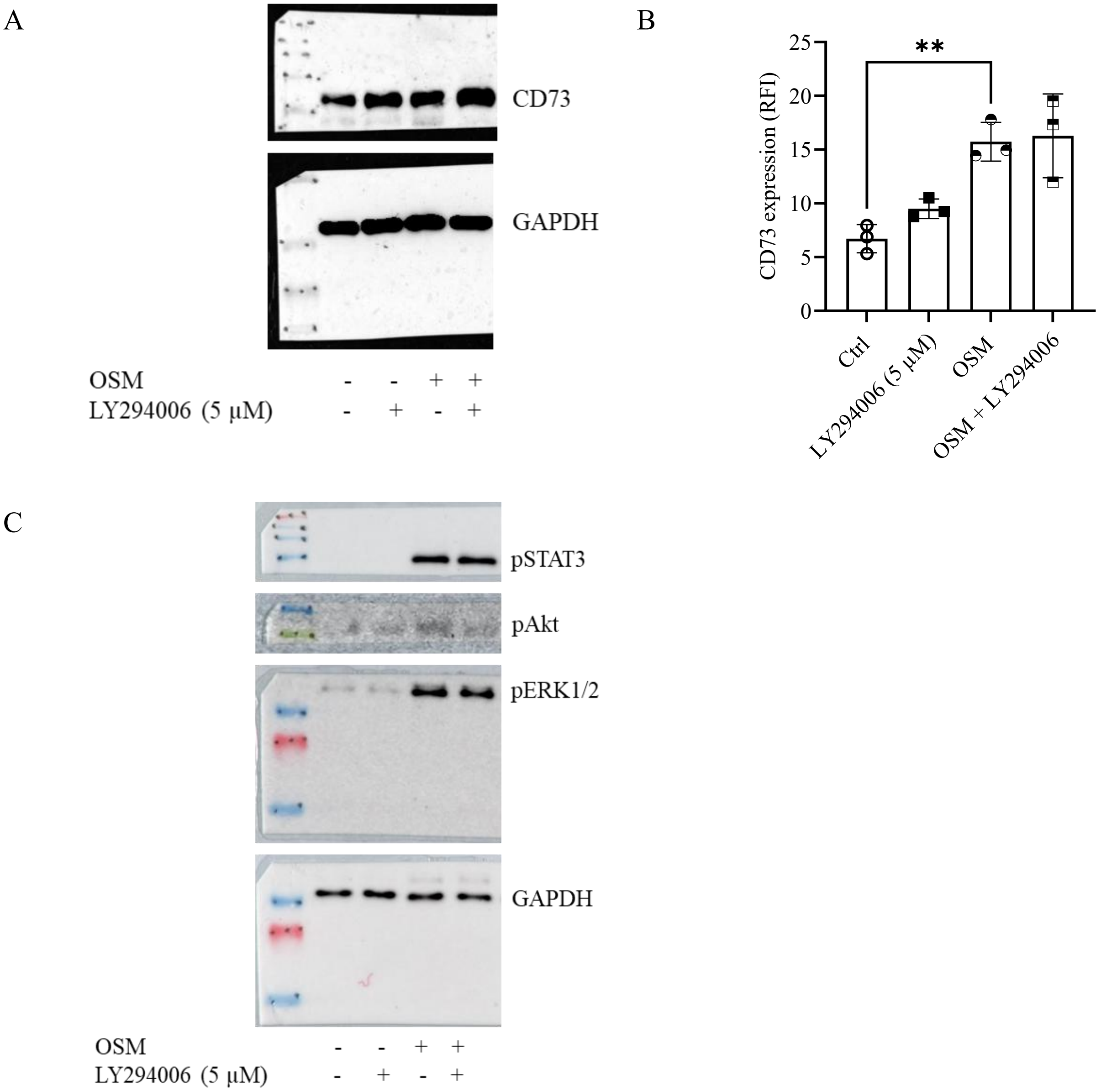

Supplementary figure 5. (A) Representative blot showing the expression of CD73 in cells treated with the PI3K pathway inhibitor LY-294006 (5 µM) 15 min before stimulation with OSM (10 ng/mL) for 48 h. (B) Expression of CD73 determined by flow cytometry in keratinocytes treated with LY294006 (5 µM) 2 h before adding OSM (10 ng/mL) for 48 h. Data are mean ± s.d. as RFI, n = 4. P value is obtained from one-way ANOVA test, followed by Šídák's multiple comparisons test. *P<0.5, compared with the indicated controls. (C) Representative blots showing the expression of phospho STAT3, phospho Akt and phospho ERK1/2 in HaCaT cells treated with the PI3K pathway inhibitor LY-294006 (5 µM) 30 min before stimulation with OSM (10 ng/mL) for 15 min. GAPDH was used as an internal control.

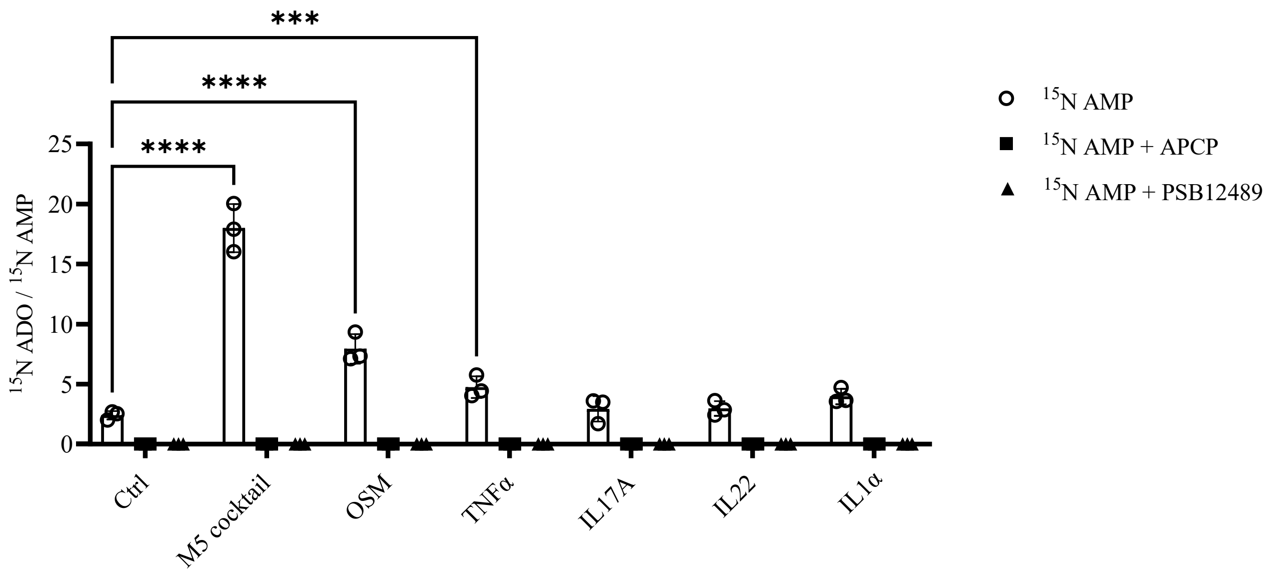

Supplementary figure 6. Ratio of [^{15}N]adenosine ([^{15}N]ADO, pg) and [^{15}N]adenosine-5'-monphosphate ([^{15}N]AMP, pg) produced by HaCaT cells in presence of the substrate [^{15}N]AMP (10 µM) upon treatment with M5 cocktail (10 ng/mL) or single cytokines (TNF-α, IL-17A, IL-22, IL-1α, OSM; 10 ng/mL) for 48 h and determined by UHPLC-ESI-MS. Selective CD73 inhibitors, APCP (100 µM) or PSB-12489 (1 µM), were also used to block the AMPase activity mediated by CD73. Data expressed are mean ± s.d., n = 3. Ctrl, control. P value is obtained from two-way ANOVA test, followed by Šídák's multiple comparisons test. **P<0.01, ***P<0.001, ****P<0.0001

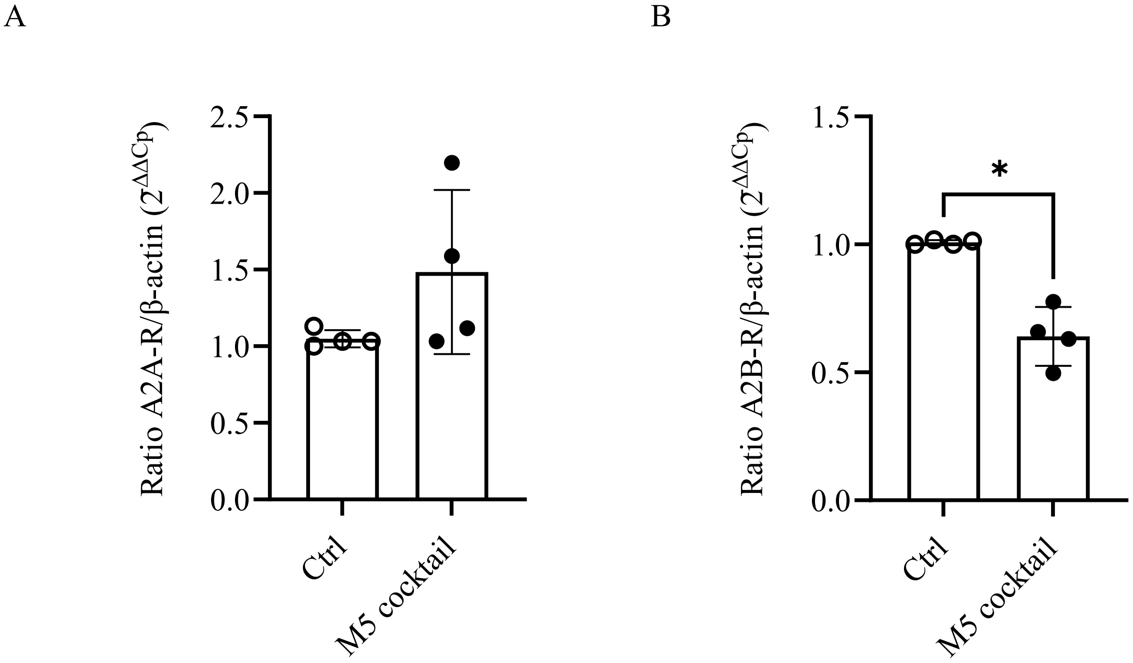

Supplementary figure 7. (A) and B) mRNA levels of A_{2A} and A_{2B} adenosine receptors, respectively, in HaCaT cells stimulated for 24 h with M5 cocktail (10 ng/mL). Data are mean ± s.d., n = 4. The relative expression level of A_{2A} and A_{2B} was normalized to those of β-actin and quantified using the $2^{-\Delta\Delta Ct}$ method. Ctrl, control. P value is obtained from a Mann Whitney test. *P<0.05.

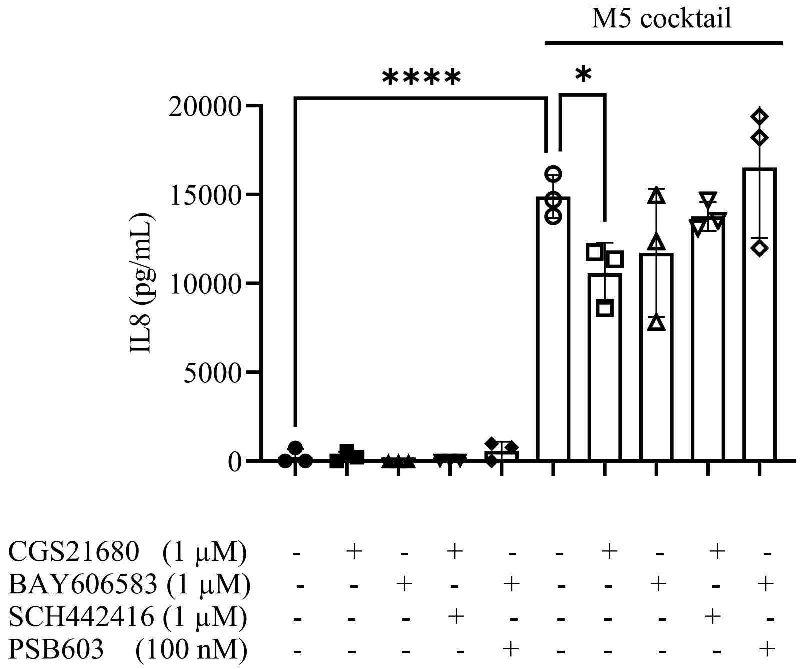

Supplementary figure 8. Levels of IL-8 measured by ELISA assay in the supernatants of HaCaT cells incubated with CGS21680 (1 μM) or BAY606583 (1 μM), alone or in combination with SCH442416 (1 μM) or PSB603 (100 nM), respectively, in the presence or absence of M5 cocktail (10 ng/mL) for 24 h. Data are mean ± s.d., n = 3. P value is obtained from one-way ANOVA test, followed by Šídák's multiple comparisons test. *P<0.05, ****P<0.0001, compared with the indicated controls.

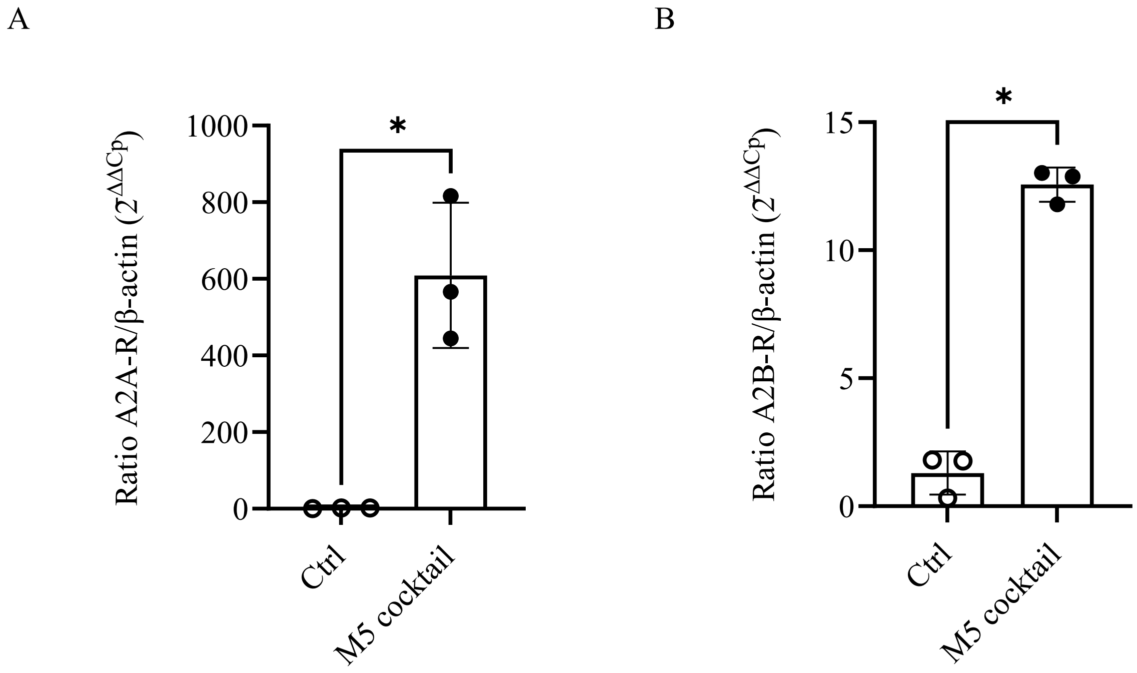

Supplementary figure 9. A) and B) mRNA levels of A_{2A} receptor and A_{2B} receptor, respectively, in dermal fibroblasts BJ stimulated with M5 cocktail (10 ng/mL) for 24 h. Data are mean ± s.d., n = 3. The relative expression level of A_{2A} and A_{2B} receptors was normalized to those of β-actin and quantified using the $2^{-\Delta\Delta Ct}$ method. Ctrl, control. P value is obtained from a Mann Whitney test. *P<0,05 compared with control.

ORIGINAL UNCROPPED WESTERN BLOT IMAGES

Figure 1E

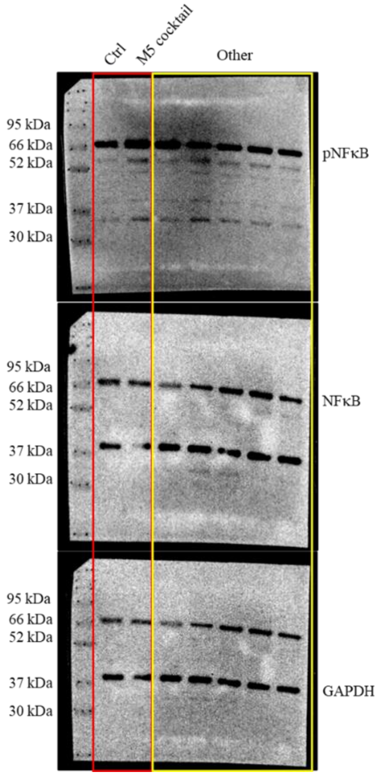

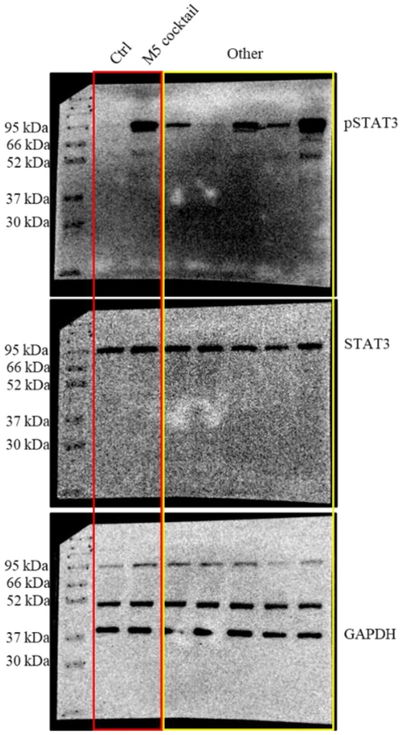

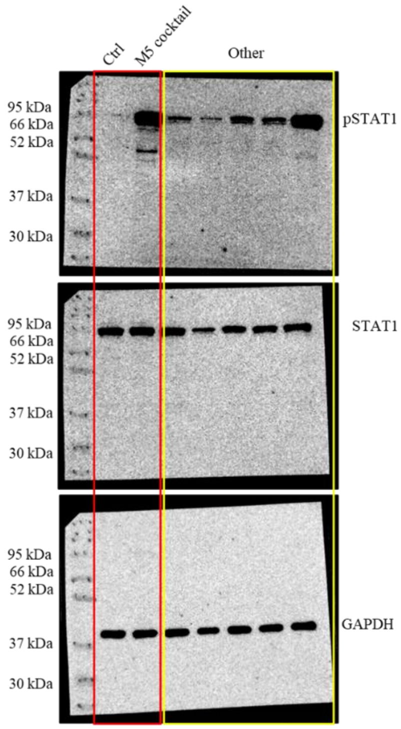

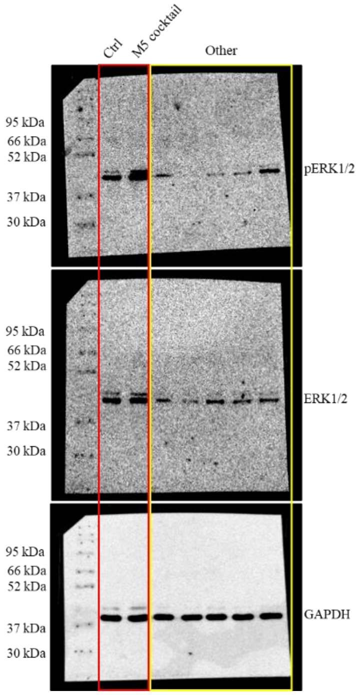

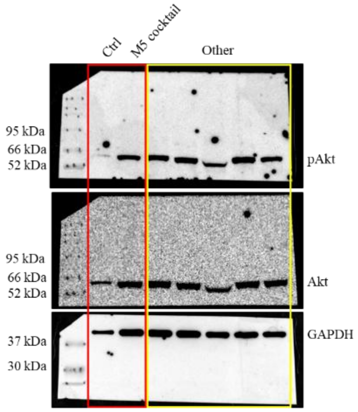

Figure 1J

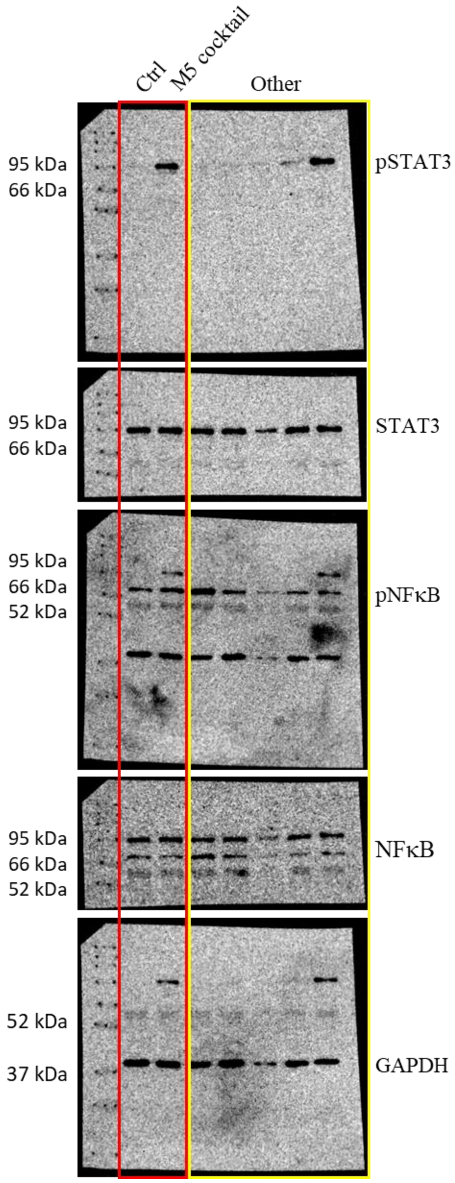
Ctrl
M5 cocktail
Other
95 kDa
66 kDa
pSTAT3
95 kDa
66 kDa
STAT3
95 kDa
66 kDa
52 kDa
pNFκB
95 kDa
66 kDa
52 kDa
NFκB
52 kDa
37 kDa
GAPDH

Figure 2B

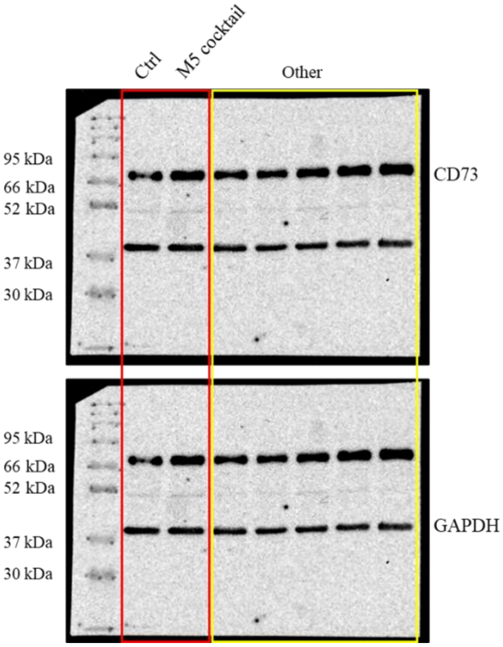

Figure 3B

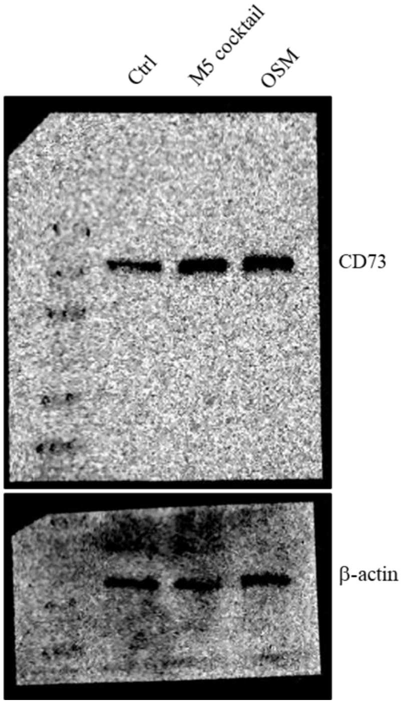

Figure 4B

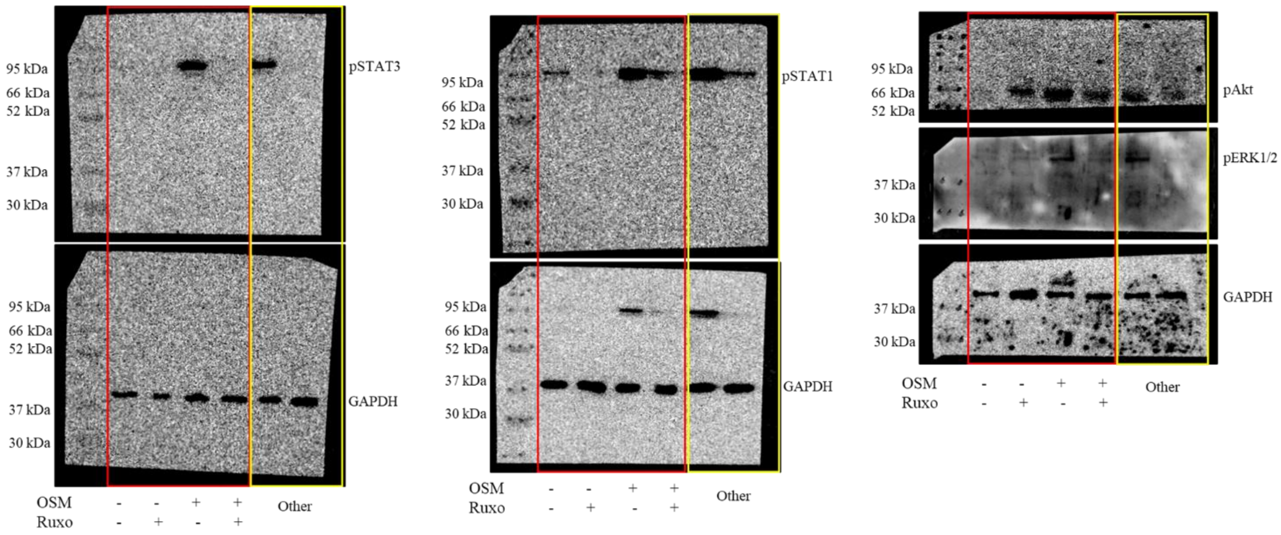

Figure 5A

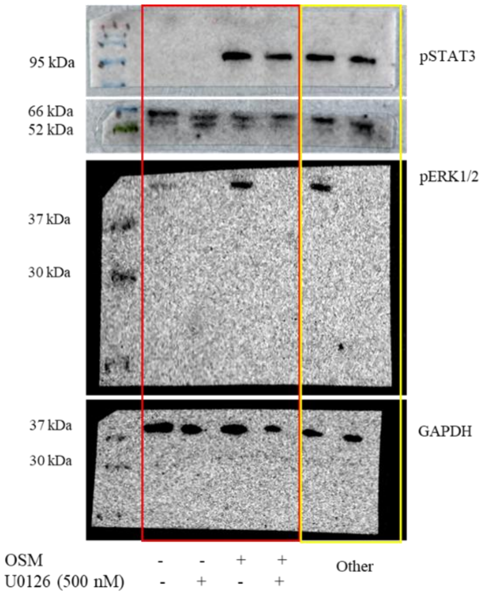

Figure 5C

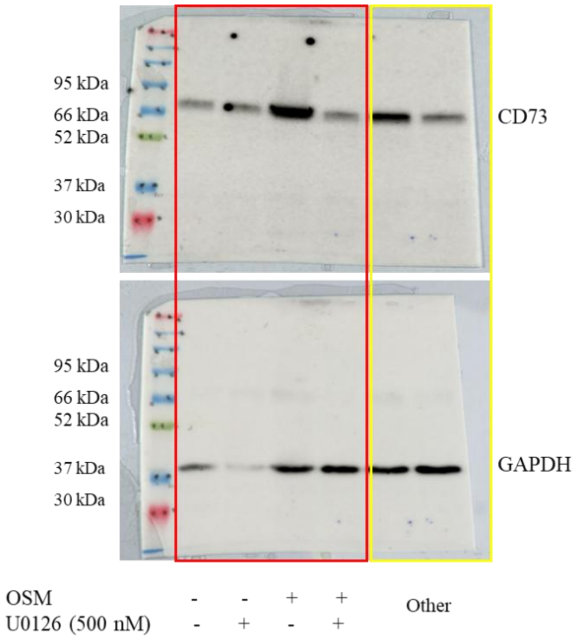

Figure 5D

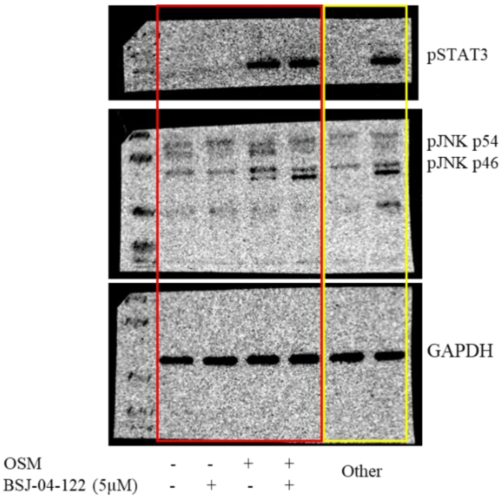

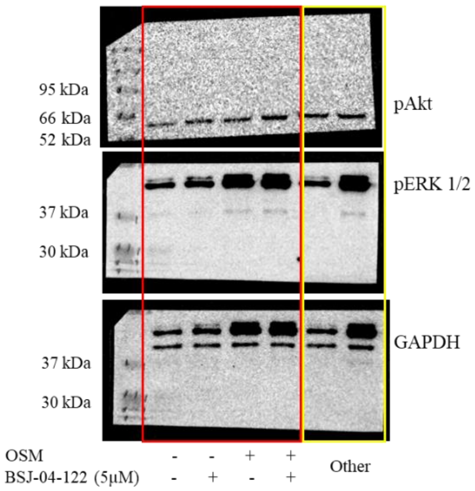

Figure 7B

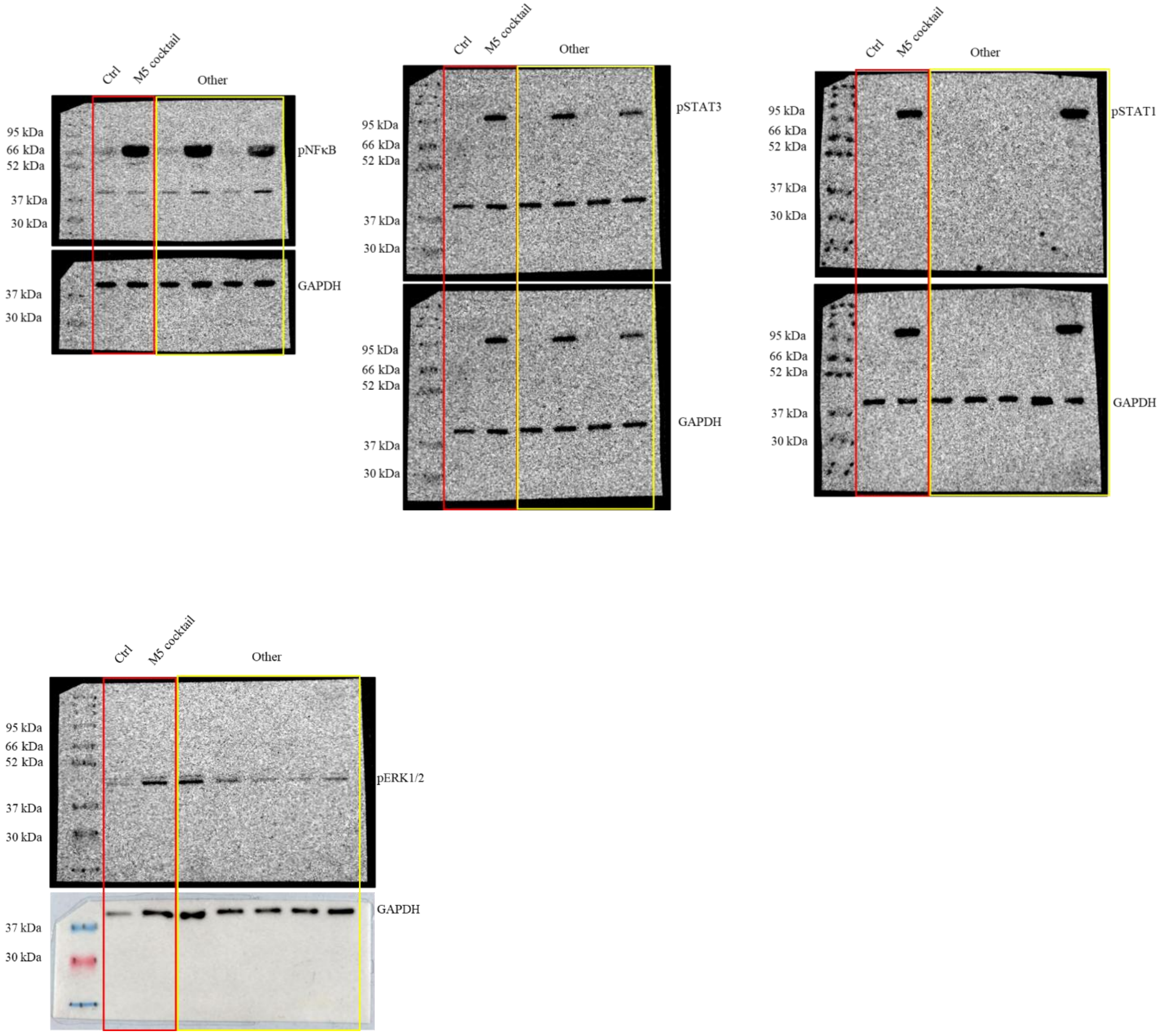
Ctrl
M5 cocktail
Other
95 kDa
66 kDa
52 kDa
37 kDa
30 kDa
pNFκB
GAPDH
Ctrl
M5 cocktail
Other
pSTAT3
95 kDa
66 kDa
52 kDa
37 kDa
30 kDa
GAPDH
Ctrl
M5 cocktail
Other
pSTAT1
95 kDa
66 kDa
52 kDa
37 kDa
30 kDa
GAPDH
Ctrl
M5 cocktail
Other
95 kDa
66 kDa
52 kDa
37 kDa
30 kDa
pERK1/2
GAPDH
